# Antimicrobial peptide-producing dermal preadipocytes defend against *Candida albicans* skin infection via the FGFR-MEK-ERK pathway

**DOI:** 10.1371/journal.ppat.1011754

**Published:** 2023-11-30

**Authors:** Jianing Wang, Zhimin Duan, Rong Zeng, Lu Yang, Weizhao Liu, Yiman Liu, Qian Yao, Xu Chen, Ling-juan Zhang, Min Li

**Affiliations:** 1 Jiangsu Key Laboratory of Molecular Biology for Skin Diseases and Sexually Transmitted Infections, Institute of Dermatology, Chinese Academy of Medical Sciences and Peking Union Medical College, Nanjing, China; 2 School of Public Health, Nanjing Medical University, Nanjing, China; 3 State Key Laboratory of Cellular Stress Biology, School of Pharmaceutical Sciences, Xiamen University, Xiamen, China; Memorial Sloan-Kettering Cancer Center, UNITED STATES

## Abstract

Dermal fibroblasts (dFBs) defend against deep bacterial skin infections by differentiating into preadipocytes (pAds) that produce the antimicrobial peptide cathelicidin; this differentiation is known as the dermal reactive adipogenesis response. However, the role of dFBs in fungal infection remains unknown. Here, we found that cathelicidin-producing pAds were present in high numbers in skin lesions from patients with cutaneous *Candida* granulomas. Second, we showed that dermal *Candida albicans* (*C*. *albicans*) infection in mice robustly triggered the dermal reactive adipogenesis response and induced cathelicidin expression, and inhibition of adipogenesis with pharmacological inhibitors of peroxisome proliferator–activated receptor γ (PPARγ) impaired skin resistance to *C*. *albicans*. *In vitro*, *C*. *albicans* products induced cathelicidin expression in pAds, and differentiating pAds markedly suppressed the growth of *C*. *albicans* by producing cathelicidin. Finally, we showed that *C*. *albicans* induced an antimicrobial response in pAds through the FGFR-MEK-ERK pathway. Together, our data reveal a previously unknown role of dFBs in the defense against skin infection caused by *C*. *albicans*.

## Author summary

*Candida albicans* (*C*. *albicans*) is the pathogen that most commonly causes human fungal infections. It normally inhabits the skin surface, genital and/or intestinal mucosa of almost 30–70% healthy individuals. In certain instances, such as impairment of skin barrier integrity or immunosuppression, *C*. *albicans* can cause mucosal and cutaneous candidiasis. Here, we demonstrated the protective role of dermal fibroblasts against *C*. *albicans* skin infection, which is previously unknown. Firstly, we found that the number of cathelicidin-producing preadipocytes (pAds) increased in both human and mouse skin lesions infected with *C*. *albicans*, indicating that *C*. *albicans* infection triggers a dermal reactive adipogenesis response. Furthermore, we found inhibiting adipogenesis by administration of pharmacological inhibitors of peroxisome proliferator–activated receptor γ, or suppressing cathelicidin expression, impaired skin resistance to *C*. *albicans*. Finally, we showed that pAds produced cathelicidin through the FGFR-MEK-ERK pathway in response to *C*. *albicans* infection. Our results could provide new therapeutic strategies for the treatment of deep cutaneous candidiasis.

## Introduction

*Candida albicans* (*C*. *albicans*) is the pathogen that most commonly causes human fungal infections [1]. It is an opportunistic fungus and normally inhabits the skin surface of healthy individuals [1, 2]. Superficial *C*. *albicans* infections, including mucosal candidiasis or diaper rash, are common, and *C*. *albicans* can also cause deep cutaneous candidiasis [3, 4]. Immunosuppression is the main risk factor for deep cutaneous candidiasis, but immunocompetent individuals can be affected as well [1–5]. Determining the mechanisms underlying the skin’s host defense response against *C*. *albicans* invasion is crucial for infection control.

In many cases, *C*. *albicans* infection is caused by a burst of fungi into the epithelium [1, 6]. Keratinocytes, which are the predominant cell type in the epidermis, secrete cathelicidin LL-37 and β-defensin to defend against *C*. *albicans* infection [7]. Under disease conditions, *C*. *albicans* can penetrate the skin and reach the deep dermis via forming hyphae. Myeloid and lymphoid cells, such as macrophages, neutrophils and T lymphocytes, have been observed to mediate host immunity against *C*. *albicans* [8–10]. Antimicrobial peptides including cathelidicin LL-37 are important effector molecules produced by host cells, possessing a high activity against *C*. *albicans* [11, 12]. However, the roles of dermal fibroblasts (dFBs) in the defense against *C*. *albicans* infection are poorly understood. Previous studies have shown that *C*. *albicans* infection induces the expression of mouse cathelicidin mCRAMP in the dermis [13]. However, whether dFBs participate in this process remains unknown.

Recent studies have revealed that specific subpopulation of dFBs can differentiate into adipocytes upon stimulation with *Staphylococcus aureus* (*S*. *aureus*). This process is defined as reactive adipogenesis. Cathelicidin antimicrobial peptide (*Camp*) is expressed at high levels during this process and inhibits bacterial growth, as shown by the fact that inhibition of adipogenesis or knockout of *Camp* leads to higher bacterial loads and larger wound sizes in mouse skin [14]. Upon bacterial infection, dFBs commit and differentiate into PREF1+ adipogenic dFBs (preadipocytes, pAds), proliferate and initiate reactive adipogenesis [14, 15]. In addition, adipocytes derived from dermal white adipose tissue (dWAT) express higher levels of *Camp* than adipocytes from other WAT depots, such as subcutaneous WAT and epidydimal WAT, indicating that the ability to produce antimicrobial peptide is a unique feature of dWAT [15, 16]. Together, these emerging evidences indicate that dFBs have important antimicrobial immune functions.

In this study, we investigated the role of fibroblasts in *C*. *albicans* dermal infection. We first analyzed the presence of dermal pAds and the expression of antimicrobial peptide cathelicidin in skin lesions from human patients with cutaneous *Candida* granulomas and in control skin samples. Then, we established a mouse model of *C*. *albicans* skin infection and characterized how *C*. *albicans* triggered dermal reactive adipogenesis. Next, we investigated how inhibition of adipogenesis altered skin resistance to *C*. *albicans* infection. Finally, we explored the mechanisms by which *C*. *albicans* induced the expression of cathelicidin.

## Results

### Adipogenesis and cathelicidin expression are elevated in human cutaneous *Candida* granulomas

**Fig 1:**
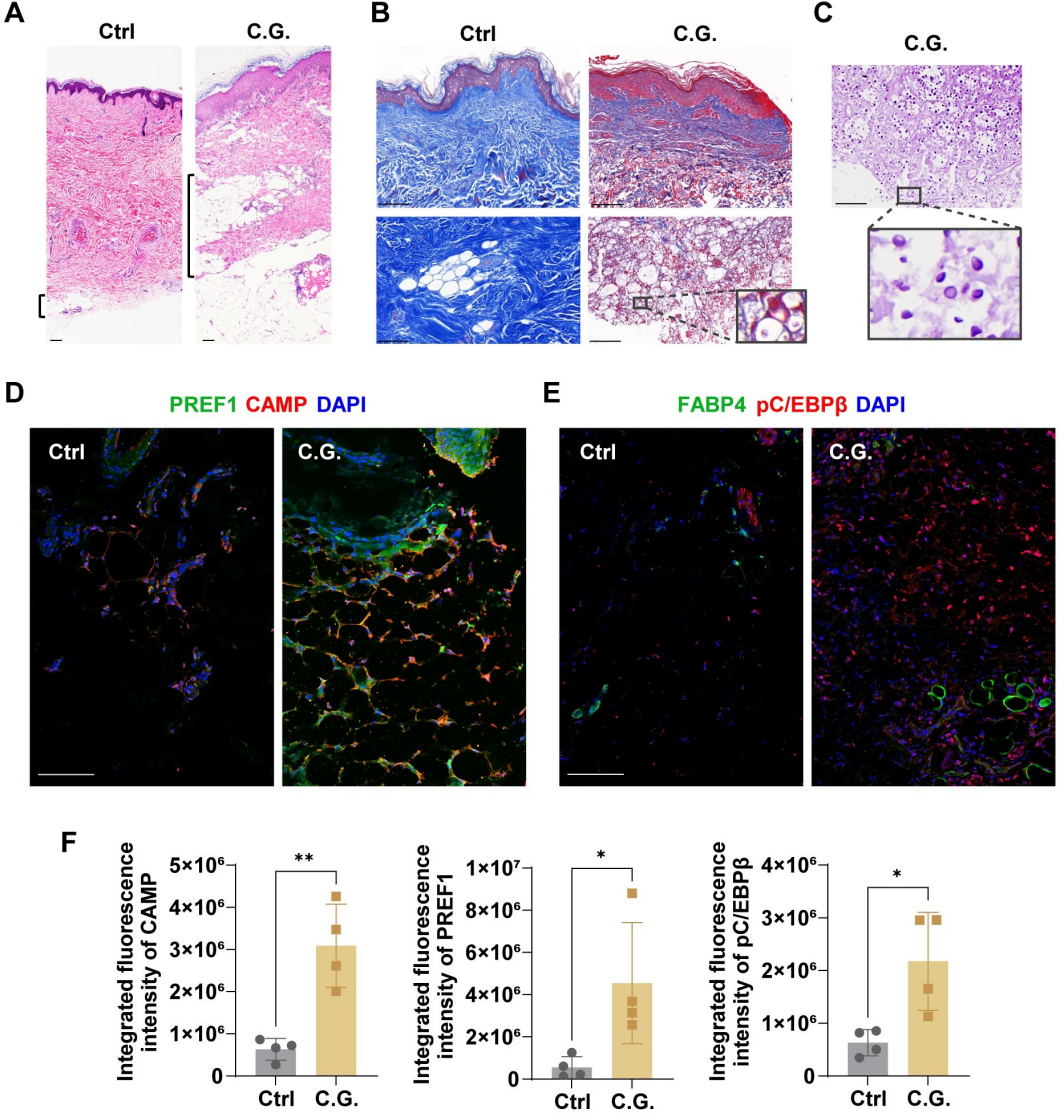
Adipogenesis and cathelicidin expression are elevated in human cutaneous *Candida* granulomas. (A and B) Hematoxylin and eosin (A) or collagen trichrome staining (B) of human skin sections from patients with cutaneous *Candida* granulomas (C.G.) and nonlesional control skin. (C) Periodic acid–Schiff (PAS) staining of *C*. *albicans* in human cutaneous *Candida* granulomas. (D–F) Immunostaining of PREF1 (green) and CAMP (red) (D) or FABP4 (green) and pC/EBPβ (red) (E) in human skin and quantification of the integrated fluorescence intensity as indicated (F). All scale bars, 100 μm. All error bars indicate mean ± SD. *P < 0.05, **P < 0.01, ***P < 0.001 (t test).

Previous studies have shown that skin bacterial infection triggers fibroblast differentiation into adipocytes [14]. To explore whether this reactive adipogenesis occurred in the dermal defense response to *C*. *albicans*, we analyzed the expression of cathelicidin (CAMP), PREF1 (pAd marker), phospho-C/EBPβ (key adipogenesis transcription factor) and FABP4 (mature adipocyte marker) [14] in skin samples from patients with cutaneous *Candida* granulomas and in nonlesional control skin tissues. Cutaneous *C*. *albicans* infection was confirmed by fungal culture of clinical skin samples. We found that besides the granulomatous inflammation caused by *C*. *albicans*, there was an increase in dWAT thickness and a reduction in collagen content in patient skin lesions compared to the control skin tissues (Fig 1A and 1B). Moreover, periodic acid–Schiff (PAS) staining assay showed that PAS-stained *C*. *albicans* yeasts were present in dWAT areas, suggesting that a potential interaction between adipocytes and *C*. *albicans* may occur in cutaneous *Candida* granulomas (Fig 1C). Moreover, immunofluorescence staining assays showed that the protein levels of adipogenic markers (PREF1, FABP4, and pC/EBPβ [17, 18]) and cathelicidin were increased in skin lesions from the patients compared to the controls (Fig 1D–1F). These data suggest that reactive adipogenesis may take place in cutaneous *Candida* granulomas to fight against *C*. *albicans* via the expression of cathelicidin.

### *C*. *albicans* infection increases dermal adipocyte numbers in mouse skin tissue

**Fig 2:**
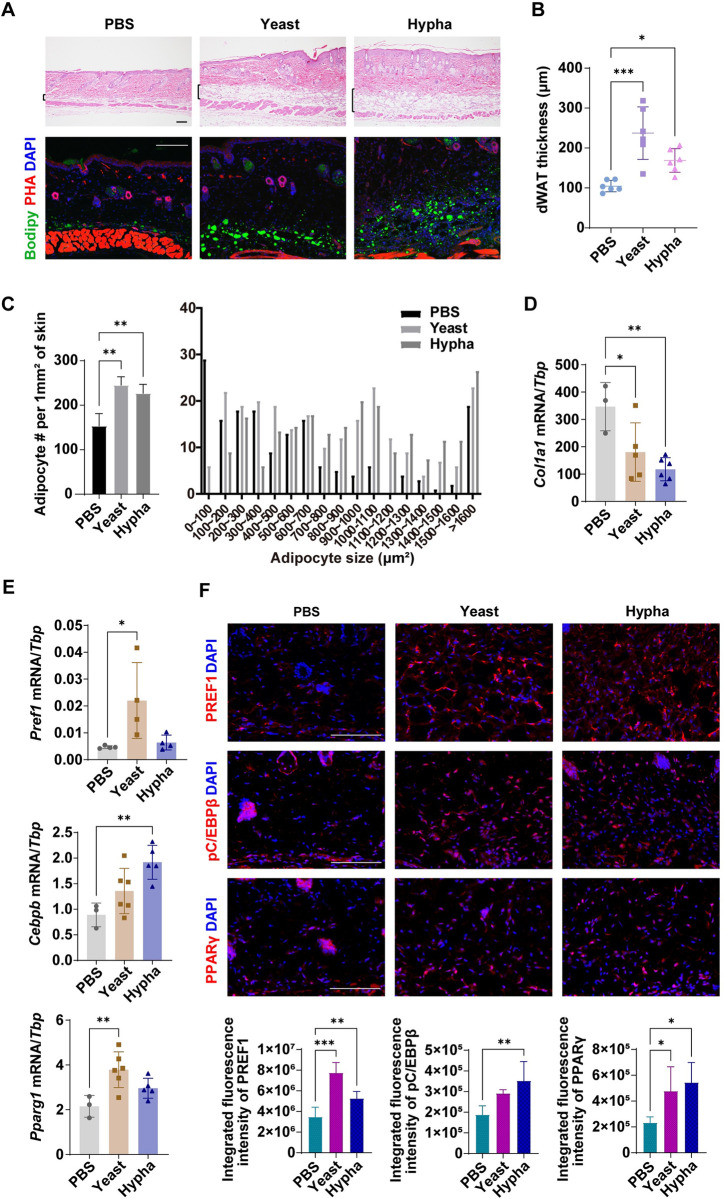
*C*. *albicans* infection increases dermal adipocyte numbers in mouse skin tissue. Mouse skin were intradermally injected with PBS control or *C*. *albicans* yeasts or hyphae, skin samples were collected on day 3. (A) Hematoxylin and eosin (top) or BODIPY staining (bottom) of mouse skin. Nuclei were stained with DAPI. Brackets indicate dWAT. Scale bars, 100 μm. (B) The DWAT thickness of mouse skin as indicated (n = 6/group). (C) Quantification of the number and size of perilipin-1^+^ adipocytes. (D and E) The mRNA expression of *Col1a1* (D), *Pref1*, *Cebpb* and *Pparg1* (E) in mouse skin (n = 3~6/group). (F) Immunostaining of PREF1, pC/EBPβ and PPARγ in PBS vehicle or *C*. *albicans*-injected skin (top) and quantification of the integrated fluorescence intensity as indicated (bottom). Scale bars, 100 μm. All error bars indicate mean ± SD. *P < 0.05, **P < 0.01, ***P < 0.001 (one-way ANOVA). dWAT, dermal white adipose tissue; PBS, phosphate buffered saline; PHA, phalloidin.

To further verify whether adipogenesis occurs during *C*. *albicans* infection, we established a mouse model of *C*. *albicans* skin infection in which mice were infected with either *C*. *albicans* yeasts or hyphae. Granulomatous inflammation was observed around the inoculation site (S1A Fig) and dermal infection was confirmed by PAS staining (S1B Fig). Consistent with the observations from the cutaneous granuloma patients, we observed that the dWAT thickness and lipid droplet content were increased in the infected area of infected mice compared with control mice, according to H&E and BODIPY staining assays (Figs 2A, 2B, and S1C–S1E). Moreover, the number and size of the adipocytes in the dermis were increased after infection (Fig 2C). The mRNA levels of *Col1a1* (Fig 2D) and the collagen content were decreased in the skin lesions of *C*. *albicans*-infected mice (S1F Fig), indicating that the fibrotic features of the dermis were suppressed after infection [15]. The mRNA levels of *Pref1*, *Cebpb* and *Pparg* (three adipogenic markers) were increased after *C*. *albicans* infection (Fig 2E). By immunostaining assay, we confirmed that the protein levels of PREF1, phospho-C/EBPβ and PPARγ were increased in the infected area (Fig 2F). These data suggest that *C*. *albicans* infection triggers a fibroblast adipogenesis response in the skin dermis.

### Adipogenesis-related PPARγ activity in infected skin may be crucial to counteract *C*. *albicans* infection

**Fig 3:**
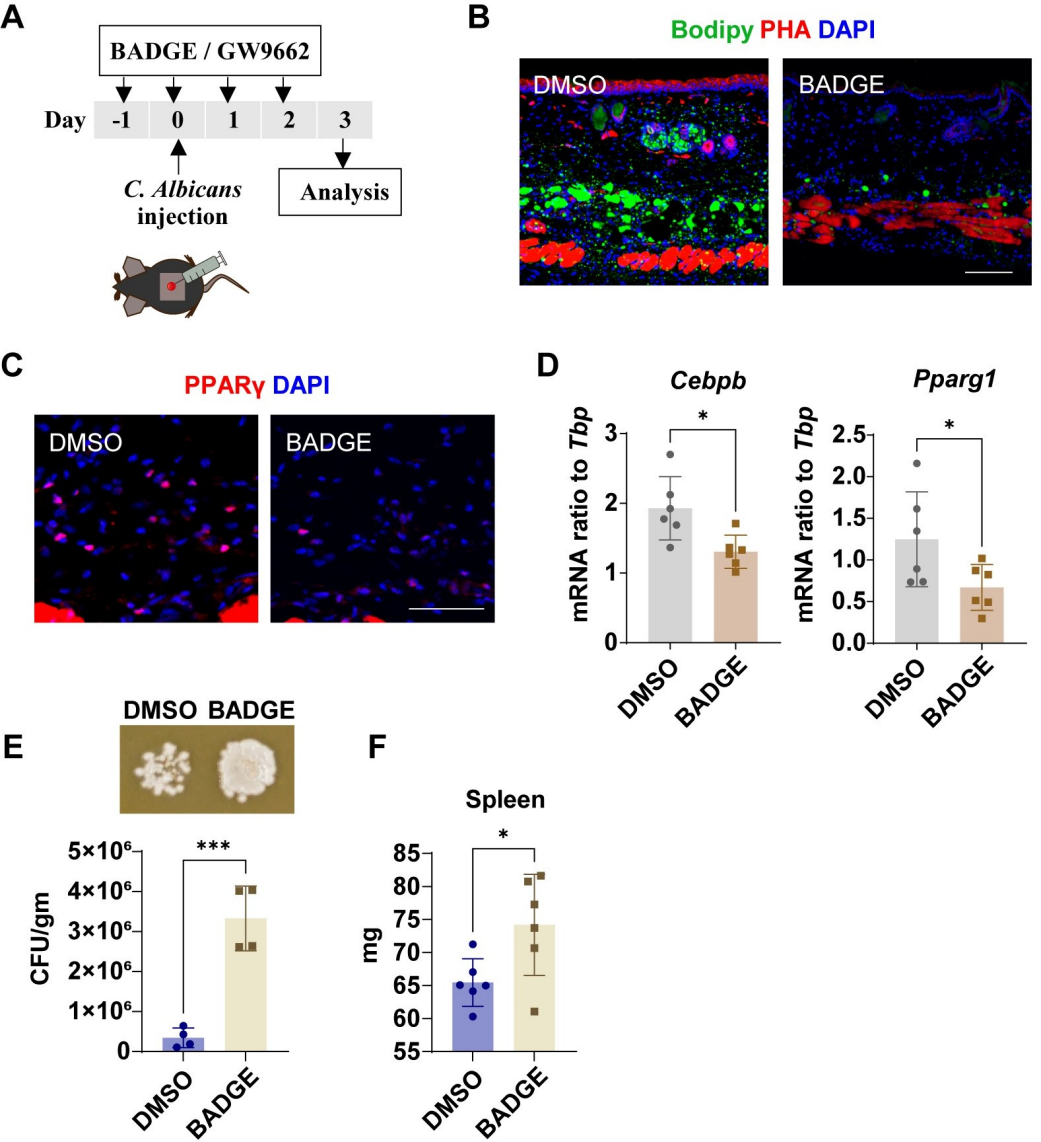
Adipogenesis-related PPARγ activity in infected skin may be crucial to counteract *C*. *albicans* infection. (A) Schematic of BADGE or GW9662 daily intraperitoneal injection, starting 1 day before infection. Skin samples were analyzed on day 3. (B) BODIPY staining of infected mouse skin with the treatment of DMSO control or BADGE. Scale bar, 100 μm. (C) Immunostaining of PPARγ in *C*. *albicans*-infected skin as indicated. Scale bar, 50 μm. (D) The mRNA expression of *Cebpb* and *Pparg1* in mouse skin (n = 6/group). (E and F) CFU analysis of *C*. *albicans*-infected skin lesions and the growth image of *C*. *albicans* on SDA plate for 36 h (E) and the weight of spleen as indicated (F) (n = 4~6/group). All error bars indicate mean ± SD. *P < 0.05, **P < 0.01, ***P < 0.001 (t test). CFU, colony-forming unit; SDA, sabouraud dextrose agar.

Peroxisome proliferator–activated receptor γ (PPARγ) is the key transcription factor that regulates adipogenesis [14, 19, 20]. To investigate the role of reactive adipogenesis during *C*. *albicans*-induced skin infection, infected mice were treated with the PPARγ pharmacological inhibitor bisphenol A diglycidyl ether (BADGE) to suppress adipogenesis (Fig 3A) [19, 21]. On the 3^rd^ day after *C*. *albicans* challenge, we observed that adipose expansion was reduced in BADGE-treated mice (Fig 3B). Through an immunofluorescence staining assay, we confirmed that BADGE treatment effectively decreased PPARγ expression (Fig 3C) and the mRNA levels of *Cebpb* and *Pparg* in the infected area (Figs 3D and S2A). Considering that skin bacteria can modulate PPARγ signaling [22, 23], we determined the amounts of skin bacteria in DMSO (control) and BADGE-treated mice [24]. We found that there was no significant difference in the expression of *16S rRNA* between the two groups (S2B Fig). *S*. *aureus*-infected mouse skin was used as a positive control (S2C Fig). Importantly, we found that BADGE treatment led to increased numbers of fungal colony-forming units (CFUs) in the skin lesion and increased spleen weight (Fig 3E and 3F). Similar CFU quantification results were observed in GW9662 (another PPARγ inhibitor)-treated mice (S2D Fig), although there was no significant difference in the skin lesion size between control and two groups of PPARγ inhibitor-treated mice (S2E Fig). These results suggest that suppression of PPARγ signaling results in increased susceptibility to cutaneous *C*. *albicans* infection.

### Cathelicidin produced by pAds protects against *C*. *albicans* infection

**Fig 4:**
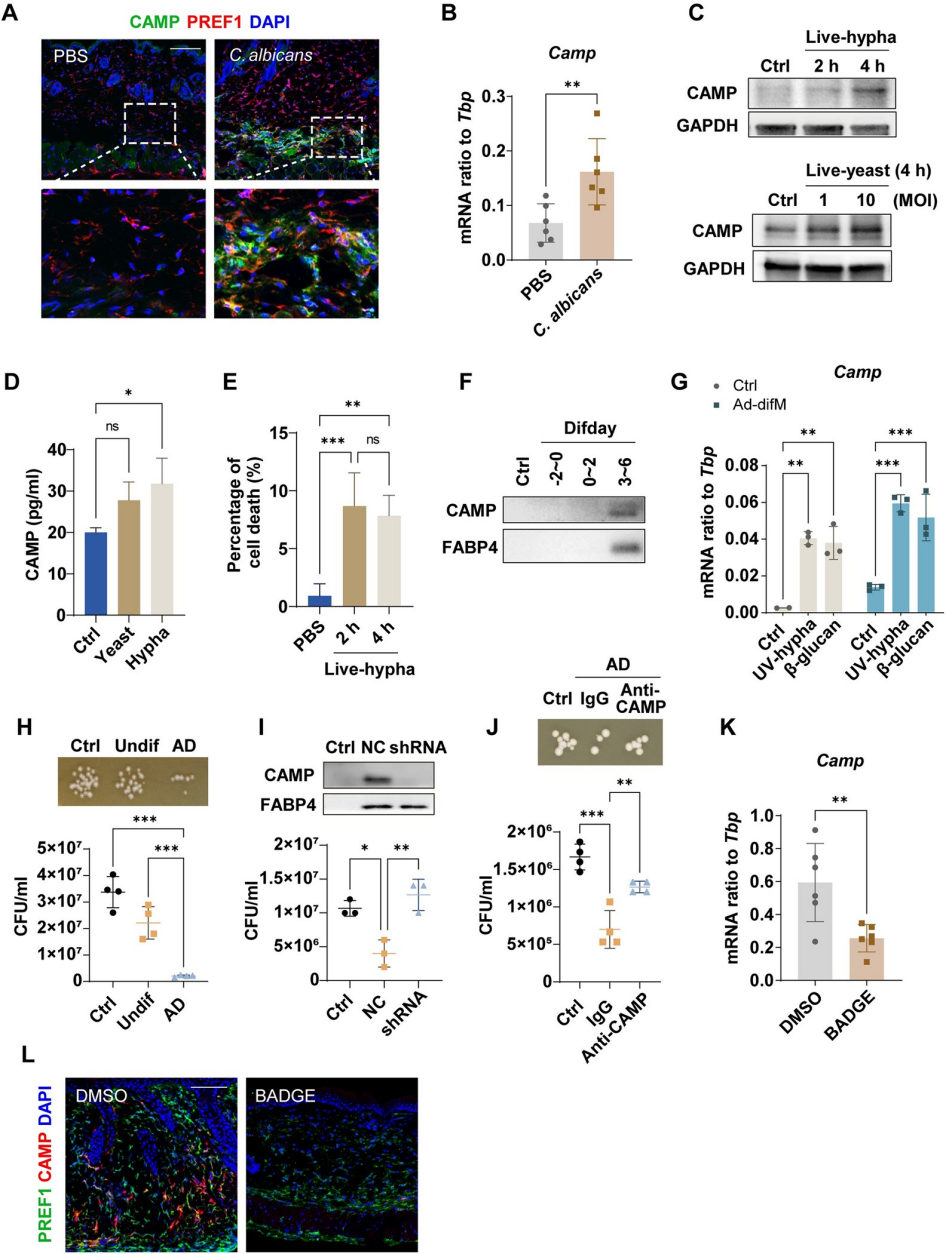
Cathelicidin produced by pAds protects against *C*. *albicans* infection. (A and B) Enhanced cathelicidin (CAMP) expression in *C*. *albicans*-infected mouse skin was shown by immunostaining of CAMP (green) and PREF1 (red) (A) or *Camp* mRNA expression (n = 6/group) (B). Scale bar, 100 μm. (C–G) Neonatal dermal fibroblasts (dFBs) were cultured to reach 100% confluence (Difday-2) for 2 days (Difday0, preadipocytes, pAds), then stimulated with PBS control (Ctrl) or *C*. *albicans*. (C) Western blotting analysis of CAMP protein level as indicated (MOI = 10 for hyphae, stimulated for 2 or 4 h; MOI = 1 and 10 for yeasts, stimulated for 4 h). (D) Preadipocytes were stimulated with *C*. *albicans* live yeasts (MOI = 1) or hyphae (MOI = 0.1) for 1 day. CAMP levels were analyzed by ELISA. (E) Percentage of cell death after stimulation with hyphae (MOI = 10) was determined by lactate dehydrogenase (LDH) assay as indicated. (F) Adipocyte transformation was triggered in pAds through treating with adipocyte differentiation medium (Ad-difM) from Difday0. 20 μL of conditioned medium per sample (Difday-2~0, Difday0~2 and Difday3~6) was used for western blotting analyses. Western blotting analysis of CAMP and FABP4 protein in conditioned medium (CM) or culture medium (Ctrl) as indicated. (G) The mRNA expression of *Camp* induced by UV-killed hyphae or β-glucan in undifferentiated or differentiating pAds after 4 h of stimulation (n = 3). (H) Growth image of *C*. *albicans* on SDA plate (top) or CFU count of *C*. *albicans* cultured in CM from culture medium (Ctrl), undifferentiated pAds (Undif) or differentiating adipocytes (AD) at 20 h (bottom). (I and J) CM was collected from differentiating adipocytes or culture medium (Ctrl). (I) Western blotting analysis of CAMP and FABP4 protein in CM (top) or CFU count of *C*. *albicans* cultured in CM (bottom) after depletion of *Camp* by shRNA in pAds. (J) CAMP neutralizing antibody (5 μg/ml) or a mouse IgG control was added to CM. Growth image of *C*. *albicans* on SDA plate (top) or CFU count of *C*. *albicans* cultured in CM at 30 h as indicated (bottom). (K and L) Loss of *Camp* expression in *C*. *albicans*-infected mouse skin after BADGE application, shown by mRNA expression (K) or immunostaining (L). Scale bar, 100 μm. All error bars indicate mean ± SD. *P < 0.05, **P < 0.01, ***P < 0.001 (Unpaired t test was used in B and K, one-way ANOVA multiple comparison test was used to determine statistical significance in D, E, H–J). Difday, differentiation day.

Studies have shown that cathelicidin is secreted at high levels during adipogenesis [14, 15]. As an antimicrobial peptide, cathelicidin exhibits powerful microbial killing activity [25]. To explore whether cathelicidin was produced and participated in the protection against *C*. *albicans* infection during adipogenesis, we first examined the expression of cathelicidin in the skin of *C*. *albicans*-infected mice. By immunostaining assay, we found that the protein level of cathelicidin was increased in the infected area (Figs 4A and S3A). Moreover, colocalization of cathelicidin and the preadipocyte marker PREF1 was observed in the infected area (Fig 4A), suggesting that pAds produced cathelicidin in this process. The mRNA level of *Camp* (mRNA name of cathelicidin) was increased in skin tissue after *C*. *albicans* infection (Fig 4B).

To further investigate the *C*. *albicans-*induced expression of *Camp* in pAds, primary neonatal mouse dFBs with adipogenic potential were isolated and cultured *in vitro* [15, 26]. Cathelicidin protein expression was increased in pAds after stimulation with *C*. *albicans* yeasts, hyphae (the pathogenic form of *C*. *albicans* [1, 2, 6]), or *C*. *albicans* ligand β-glucan (*C*. *albicans* cell wall component) (Figs 4C and S3B). CAMP concentration in pAd culture supernatant was increased after stimulation with *C*. *albicans* (Fig 4D). To determine cell death percentage of pAds after stimulation with hyphae, lactate dehydrogenase (LDH) release assay was performed. After stimulation, the percentage of cell death was elevated. There was no significant difference in the percentage of cell death between 2 h and 4 h of stimulation (Fig 4E). To examine *Camp* expression during adipogenesis, we treated pAds with adipocyte differentiation medium (S3C Fig) and observed that cathelicidin was secreted at high levels by differentiating pAds, as shown by western blotting analysis of CAMP levels in conditioned medium (Fig 4F). The addition of *C*. *albicans* products during adipocyte differentiation further increased the *Camp* mRNA levels in differentiating pAds (Fig 4G). Moreover, conditioned medium collected from differentiating pAds potently inhibited the growth of *C*. *albicans* (Figs 4H and S3D). Knockdown of *Camp* expression by short hairpin RNA (shRNA) in pAds or neutralizing CAMP in conditioned medium attenuated the anti-*C*. *albicans* effects of differentiating pAds (Fig 4I and 4J). These results demonstrate that differentiating pAds inhibit the growth of *C*. *albicans* by producing the antimicrobial peptide cathelicidin.

We found that the mRNA level of *Camp* in skin tissue was lower in the PPARγ inhibitor BADGE-treated mice than in the control mice (Fig 4K). In addition, BADGE treatment suppressed the protein expression of cathelicidin and its colocalization with the preadipocyte marker PREF1 in infected skin (Fig 4L). These results demonstrate that cathelicidin is expressed by differentiating pAds and may contribute to defense against *C*. *albicans* infection *in vivo*.

### FGFR signaling mediates the response to *C*. *albicans* in pAds

Next, we sought to explore the mechanism by which *C*. *albicans* induced *Camp* expression in pAds. Previous studies have reported that Toll-like receptors (TLRs), including TLR2 and TLR4, and C-type lectin receptors contribute to the recognition of *C*. *albicans* [1, 27, 28]. To explore whether TLR signaling plays a role in *C*. *albicans*-induced *Camp* expression in pAds, primary neonatal dFBs were isolated from *Myd88* (a common adaptor molecule of TLRs [29])^*−/−*^ and wild-type (WT) litter-mate pups and coincubated with *C*. *albicans*. An increase in the *Camp* levels was observed in pAds from both *Myd88*^*−/−*^ and WT mice (S4A and S4B Fig). In addition, the protein levels of TLR2 and TLR4 in pAds were not elevated after stimulation with *C*. *albicans* or *C*. *albicans* ligand (S4C and S4D Fig), and dectin-1 (a C-type lectin) was barely expressed by dFBs (S4E–S4G Fig). These results indicate that TLRs or dectin-1 do not contribute to *C*. *albicans*-induced *Camp* expression in pAds.

**Fig 5:**
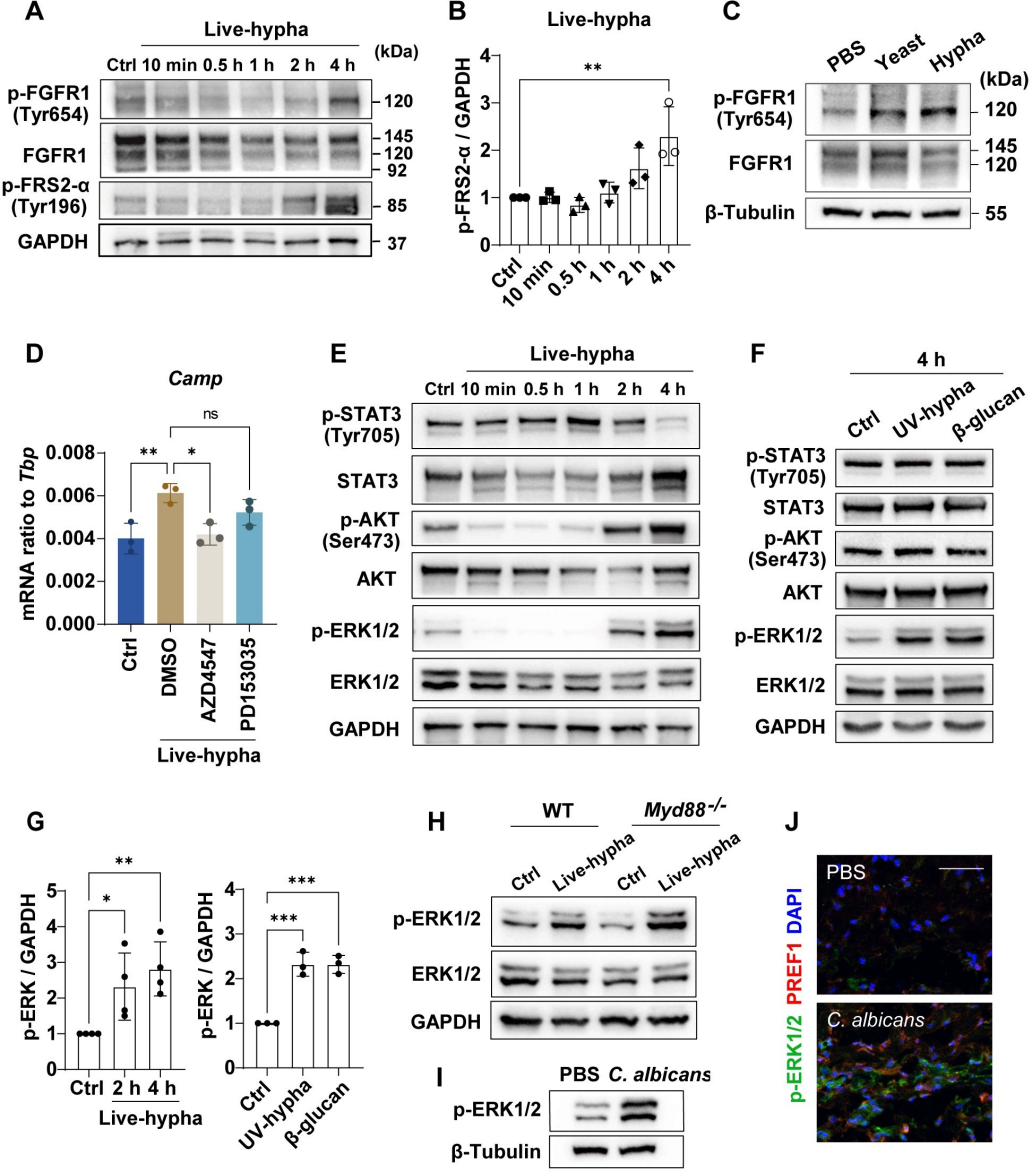
FGFR signaling mediates the response to *C*. *albicans* in pAds. (A and B) Western blotting analysis of phosphorylated FGFR1 or FRS2-α (A) and quantification of phospho-FRS2-α (B) in pAds incubated with PBS control (Ctrl) or *C*. *albicans* hyphae (MOI = 10). (C) Western blotting analysis of FGFR1 phosphorylation in PBS control or *C*. *albicans*-infected mouse skin. (D) *Camp* mRNA expression in pAds after pretreatment with PD153035, AZD4547 or vehicle control for 2 h and then stimulated with PBS control (Ctrl) or live hyphae for 12 h. (E and F) Western blotting analysis of protein levels in pAds stimulated with live hyphae (E) or UV-killed hypha or β-glucan (F). Equal volume of PBS was used as control. (G) Quantification of phospho-ERK1/2 as indicated (n = 3~4/group). (H) Western blotting analysis of ERK1/2 phosphorylation in pAds from wild-type (WT) or *Myd88*^*−/−*^ mice after stimulation with PBS control or hyphae at 4 h. (I and J) Enhanced activation of phospho-ERK1/2 in mouse skin shown by western blotting analysis (I) or immunostaining (J) on the 3^rd^ day after infection. Scale bar, 100 μm. All error bars indicate mean ± SD. *P < 0.05, **P < 0.01, ***P < 0.001 (one-way ANOVA).

Considering that fibroblast growth factor signaling is related to pathogen–host interactions [30–32], we next investigated whether fibroblast growth factor receptor (FGFR) participates in the response against *C*. *albicans* in pAds. Phosphorylation levels of FGFR1 and FGFR substrate 2α (FRS2α), the key adaptor protein of FGFRs, were elevated in pAds after treatment with live *C*. *albicans* hyphae (Fig 5A and 5B), while a milder activation of FGFR1 and FRS2α was observed after stimulation with UV-killed hyphae (S5A Fig). We also observed an increase in the FGFR1 phosphorylation (Fig 5C) and FRS2α phosphorylation (S5B Fig) levels in the *C*. *albicans*-infected skin lesions compared to uninfected control skin samples. In addition, recent studies have shown that epidermal growth factor receptor (EGFR) mediates the endocytosis of *C*. *albicans* [33, 34], and EGFR can be expressed in fibroblasts [35, 36]. Interestingly, we also observed that EGFR phosphorylation was increased in pAds upon stimulation with *C*. *albicans* (S5C and S5D Fig). However, pretreatment with the pan-FGFR inhibitor AZD4547, but not the EGFR inhibitor PD153035, suppressed *C*. *albicans*-induced cathelicidin expression in pAds (Fig 5D). These data show that FGFR mediates *C*. *albicans*-induced cathelicidin expression in pAds.

Next, we analyzed how key signaling molecules downstream of FGFR, including ERK1/2, AKT and STAT3 [30], were activated in pAds in response to *C*. *albicans* stimulation. We observed that only ERK1/2 phosphorylation was significantly induced in pAds by both *C*. *albicans* and *C*. *albicans* ligand (Fig 5E–5G). Because the MAPK and NF-κB pathways are crucial to the host immune response against *C*. *albicans* [1, 37], we also analyzed the levels of phosphorylated p38, JNK and IκBα. We found that although p38 phosphorylation was elevated in pAds stimulated with live *C*. *albicans*, none of the three molecules were activated after stimulation with UV-killed hyphae or β-glucan (S5E and S5F Fig). Furthermore, *C*. *albicans*-induced activation of ERK1/2 appeared to be unaffected in pAds isolated from *Myd88*^*−/−*^ mice compared to WT mice (Fig 5H). *C*. *albicans* infection also led to ERK1/2 activation in mouse skin (Fig 5I and 5J).

Taken together, these results show that the FGFR1-ERK signaling pathway is activated in *C*. *albicans*-challenged pAds.

### Inhibiting FGFR-MEK-ERK signaling suppresses *C*. *albicans*-induced cathelicidin production by pAds

**Fig 6:**
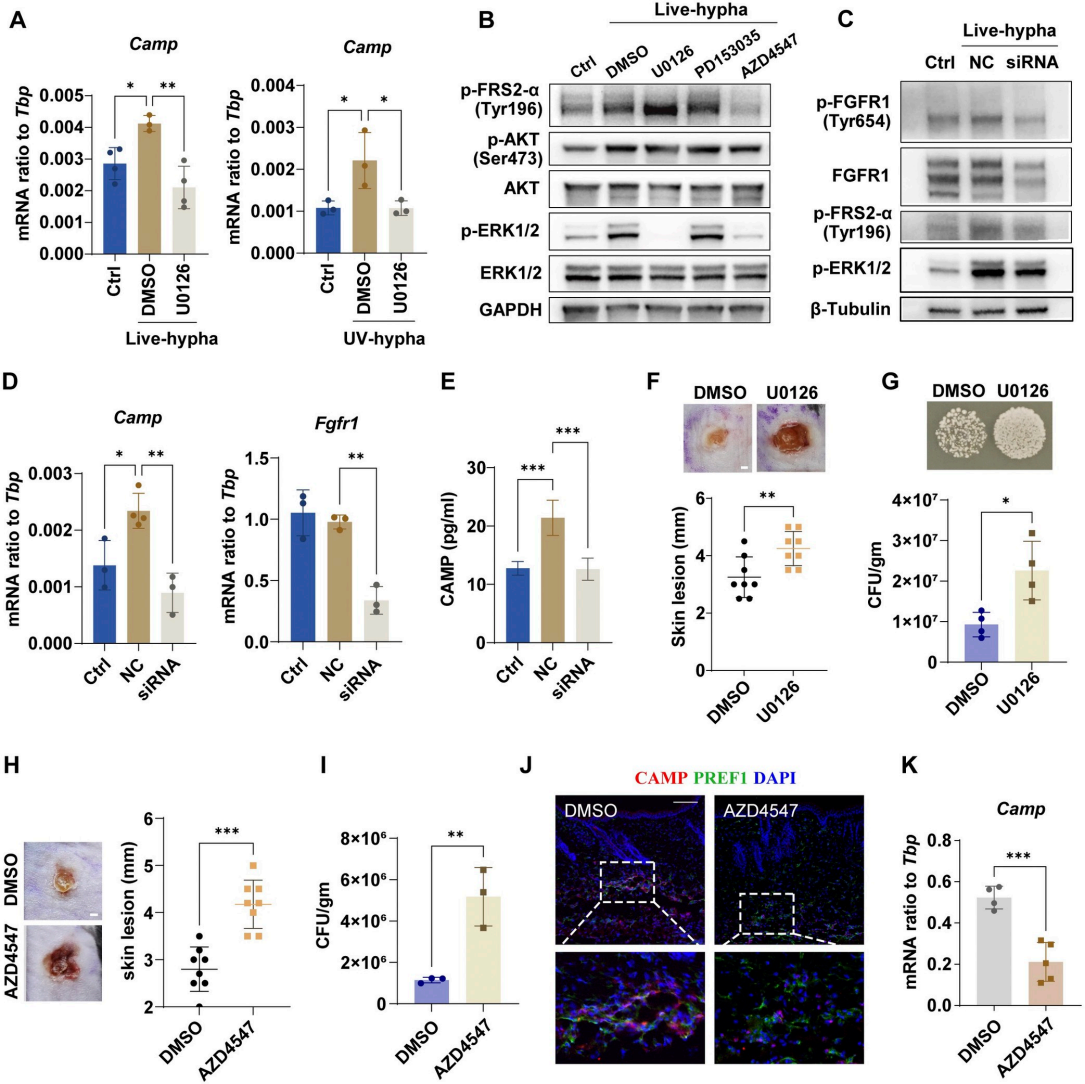
Inhibiting FGFR-MEK-ERK signaling suppresses *C*. *albicans*-induced cathelicidin production by pAds. (A) U0126 application attenuated *Camp* mRNA expression in pAds infected with *C*. *albicans* hyphae for 12 h. (B) Mouse pAds were pretreated with U0126, PD153035, AZD4547 or vehicle control (DMSO) for 2 h and then stimulated with PBS control (Ctrl) or live hyphae for 4 h. Western blotting analysis of phosphorylated FRS2-α, AKT or ERK1/2. (C–E) Mouse pAds were transfected with negative control (NC) or FGFR1 siRNA, then stimulated with *C*. *albicans* hyphae. (C) Western blotting analysis of ERK1/2 phosphorylation induced by *C*. *albicans* at 2 h. (D) *Camp* and *Fgfr1* mRNA expression in pAds after stimulation with hyphae for 18 h. (E) CAMP levels were analyzed by ELISA (n = 4/group). (F and G) Mice were intradermally injected daily with U0126 or vehicle control (DMSO) starting 20 min before infection with *C*. *albicans*. Skin samples were collected 3 days after infection. U0126 increased susceptibility to *C*. *albicans* as observed by (F) increased lesion size and (G) increased CFU count of the skin (n = 4/group). Scale bar, 1 mm. (H–K) Mice were intradermally injected daily with AZD4547 or vehicle control starting 20 min before infection with *C*. *albicans*. Skin samples were collected 3 days after infection. (H) Infected lesion size and (I) CFU of *C*. *albicans* from skin lesions calculated with plate counting (n = 3/group). Loss of *Camp* expression in *C*. *albicans*-infected mouse skin after treatment with AZD4547, shown by immunostaining for CAMP (red) and PREF1 (green) (J), or mRNA expression (n = 4~5/group) (K). Scale bars, 1 mm (H), 100 μm (J). All error bars indicate mean ± SD. *P < 0.05, **P < 0.01, ***P < 0.001 (Unpaired t test was used in F–I and K, one-way ANOVA multiple comparison test was used to determine statistical significance in A, D and E).

To explore whether ERK signaling mediates cathelicidin production by pAds in response to *C*. *albicans*, we pretreated pAds with the MEK inhibitor U0126 for 2 h before infection. U0126 treatment attenuated the *C*. *albicans* products-mediated induction of *Camp* mRNA expression in pAds (Figs 6A and S6A). Furthermore, treatment with the pan-FGFR inhibitor AZD4547 or knockdown of FGFR1 by transfection with FGFR1-specific small interfering RNA (siRNA) inhibited ERK1/2 phosphorylation in *C*. *albicans*-stimulated pAds (Figs 6B, 6C and S6B). CAMP expression was also suppressed in pAds transfected with FGFR1 siRNA (Fig 6D and 6E). These results suggest that FGFR contributes to *C*. *albicans*-mediated activation of ERK signaling.

To further verify the role of FGFR-MEK-ERK1/2 signaling in the response of pAds to *C*. *albicans*, U0126 and AZD4547 were administered in the skin of *C*. *albicans*-infected mice respectively. We found that intradermal injection of U0126 led to enlargement of skin lesions and an increase in fungal load in the infected area, indicating that inhibition of ERK1/2 signaling increases skin susceptibility to *C*. *albicans* (Fig 6F and 6G). Moreover, immunofluorescence staining assays showed that the expression of cathelicidin in PREF1+ pAds was suppressed in *C*. *albicans*-infected skin lesions after treatment with U0126 (S6C Fig). Similar results were found in AZD4547-treated mice (Fig 6H–6K). These results demonstrate that FGFR-MEK-ERK signaling plays a key role in *C*. *albicans*-mediated reactive adipogenesis and the antimicrobial response.

Studies have reported that candidalysin, a cytolytic toxin secreted by *C*. *albicans* hyphae, induces the secretion of FGFR ligand fibroblast growth factor-2 (FGF-2) in host responses [38, 39]. To explore whether candidalysin plays a crucial role in inducing FGFR-mediated cathelicidin expression in pAds, we stimulated pAds with candidalysin *in vitro*. We observed an increase in the *Camp* mRNA levels in pAds (S6D Fig). However, we found that there was no increase in the FGFR1 phosphorylation or FRS2α phosphorylation after stimulation of candidalysin (S6E Fig), indicating that FGFR signaling was not activated by candidalysin. We also detected the level of phosphorylated EGFR in pAds as a positive control (S6F Fig). Moreover, after treatment with the FGFR inhibitor AZD4547, candidalysin-induced increase of *Camp* mRNA was not attenuated (S6G Fig) and candidalysin-induced ERK1/2 phosphorylation was unaffected (S6H Fig). Thus, candidalysin may not be the key pathogen factor of *C*. *albicans* for inducing FGFR-mediated cathelicidin production in pAds.

## Discussion

The interaction between host cells and *C*. *albicans* has been extensively investigated because of the high prevalence and severity of infection with this pathogen. However, little attention has been given to fibroblasts, the major cell type in the dermis. In this study, we explored the protective role of dFBs in *C*. *albicans* dermal infection by investigating both human cutaneous *Candida* granulomas and mouse acute infection models. Our data show that adipogenic dFBs (pAds) respond to *C*. *albicans* infection through a reactive adipogenesis response, in which the antimicrobial peptide cathelicidin is produced by activated pAds to limit the growth of *C*. *albicans*. We further demonstrate, to our knowledge, a previously unreported role of FGFR signaling in *C*. *albicans* recognition and response by pAds.

Similar responses of dFBs during bacterial skin infection have recently been reported [14, 40]. Cathelicidin production during adipogenesis contributes to protection of skin from *S*. *aureus* infection [14, 15, 26]. A recent study from Dr. Gallo’s group reported that adipogenesis in perifollicular dermal preadipocytes is essential to the pathophysiology of acne, which is primarily colonized by *Cutibacterium acnes* that is lipophilic [40]. In our study, we found that *C*. *albicans* infection promoted adipose expansion in dWAT. Inhibiting adipogenesis led to a higher fungal load in skin, and depletion of *Camp* abolished the antimicrobial effect of conditioned medium from differentiating pAds against *C*. *albicans*. These results indicate that adipogenesis and subsequent antimicrobial peptide production (cathelicidin) protect against *C*. *albicans* infection. These findings suggest that pAds play differential roles in infectious and noninfectious diseases. Compared to mice, humans lack a striated muscle in the dermis that separates dWAT from subcutaneous adipose tissue [41]. The dWAT layer in humans is considered to be located above subcutaneous adipose tissue and tends to be perifollicular [42]. Compared to nonlesional control skin samples, human cutaneous *Candida* granulomas exhibited increase in cathelicidin-expressing preadipocyte numbers in the reticular dermis and the upper part of dWAT. In addition, loss of the adipogenic ability of dFBs has been observed during aging or in obesity and was shown to be mediated by TGF-β [15, 26]. These findings may partly explain the clinical observation that aged and obese people are more vulnerable to fungal infections [43, 44]. The role of adipogenesis in the pathogenesis of cutaneous *Candida* granulomas in humans remains to be investigated further.

FGFR signaling is known to be responsible for several cellular physiological processes, including cell proliferation, migration and differentiation [30, 45, 46]. Our findings suggest that the pAd response to *C*. *albicans* is at least partially dependent on FGFR signaling. Furthermore, several studies have reported that FGFR signaling is related to virus–host cell interactions [31, 32], which coincides with our finding that FGFR could play a role in the recognition of pathogens. However, whether FGFR recognizes certain components or virulence factors of *C*. *albicans* is still unknown. Previous studies have reported that candidalysin, the hypha-specific toxin, can induce the secretion of FGFR ligand [38]. We observed that although candidalysin induced *Camp* expression in pAds, it may not be the key driver of the FGFR-mediated cathelicidin production in pAds in our study. Phosphorylation of the adaptor FRS2α can lead to activation of downstream signaling via the MEK-ERK or PI3K-AKT pathways [30]. We found that *C*. *albicans*-induced cathelicidin expression in pAds is mediated by the MEK-ERK signaling cascade downstream of the FGFR signaling pathway. Previous studies have demonstrated that activation of MEK-ERK signaling promotes preadipocyte adipogenesis during the early phase of the differentiation process by enhancing the expression of PPARγ and C/EBPα [47, 48]. Thus, activation of FGFR-ERK signaling after *C*. *albicans* infection may contribute to adipogenesis in mouse skin.

In summary, our results show that dFBs defend against *C*. *albicans* infection through reactive adipogenesis and an antimicrobial response. Our findings may provide further insights into the treatment of cutaneous *Candida* granulomas by regulating cathelicidin expression in the dermis. Adipogenic fibroblasts that are capable of producing cathelicidin, might be a potential target for therapy.

## Materials and methods

### Ethics statement

Human skin sample collection was approved by the Institute of Dermatology, Chinese Academy of Medical Science Medical Ethics Committee (No. 2017-KY-022). All the animal experiments were approved by the Laboratory Animal Ethics Committee of the Institute of Dermatology, Chinese Academy of Medical Science (Nanjing, China).

### *C*. *albicans* preparation

*C*. *albicans* strain SC5314 was preserved in the China Medical Fungus Culture Collection Center. To generate the yeast phase, *C*. *albicans* was incubated in a rotary shaker (180 rpm) at 30°C for 12 h in yeast extract-peptone-dextrose (YPD) medium (OXOID). To generate hyphae, washed yeasts in log phase were grown in RPMI 1640 medium (Gibco) with constant rotary shaking (150 rpm) at 37°C for 6 h. Then, the yeasts or hyphae were harvested from the culture media after centrifugation, washed twice with PBS and adjusted to the proper concentration. UV-killed *C*. *albicans* was prepared by exposure to 2 doses of UV radiation (800 mJ/cm^2^) in a UV-DNA crosslinker. *C*. *albicans* was prepared fresh.

### Human specimens

Human skin samples were collected from patients who were diagnosed with cutaneous *Candida* granulomas according to clinicopathological features and fungal culture. In histology, cutaneous *Candida* granulomas presents as granulomatous inflammation. A dense infiltrate of lymphocytes, neutrophils and histiocytes can be seen in the skin lesion [49–51]. Skin biopsy specimens were cultured on Sabouraud dextrose agar media. The growing colonies were identified and only *C*. *albicans* was isolated in these samples. The control skin tissues were collected from the nonlesional skin area of patients who developed noninfectious and noninflammatory disease and underwent excisional surgery in our hospital. Sections of skin tissue embedded in paraffin were obtained from the Department of Pathology, and written informed consent was obtained from all patients before collection. Participants’ information is listed in S1 Table.

### Animal care and studies

WT C57BL/6 mice were purchased from Charles River (Beijing, China). *Myd88*^*−/−*^ C57BL/6 mice were generously provided by Professor Jiahuai Han (Xiamen University, Xiamen, Fujian, China). The backs of C57BL/6 male mice aged 7~8 weeks were shaved and chemically depilated. A total of 75 μl of 1×10^7^/ml log-phase *C*. *albicans* in the indicated form was prepared in PBS and intradermally injected [13, 14]. The PPARγ inhibitor BADGE (catalog number 15138, Sigma–Aldrich, 30 mg/kg) or GW9662 (catalog number S2915, Selleck, 1 mg/kg) was intraperitoneally administered daily 1 day before infection, and 10% DMSO in PBS (for BADGE) or 5% DMSO + 40% PEG300 in PBS (for GW9662) was used as a control. For MEK and FGFR inhibitor treatment, U0126 (catalog number S1102, Selleck, 0.064 mg in 50 μl of 2% DMSO + 40% PEG300 + PBS), AZD4547 (FGFR inhibitor, catalog number HY-13330, MedChemExpress, 0.01 mg in 50ul of 2% DMSO + 40% PEG300 + PBS) or vehicle control was intradermally injected 20 min before *C*. *albicans* infection in the same area and then administered daily. Skin biopsies were collected at Day 3 post infection. To determine the CFUs, skin samples were homogenized and serially diluted. Sabouraud dextrose agar (SDA) was used for plating and quantifying the CFUs per gram of tissue.

### Cell isolation and culture

Neonatal mouse dermal fibroblasts (WT and *Myd88*^*−/−*^) were isolated and cultured as previously described [14]. In brief, the whole skin was digested in 3 mg/ml dispase solution (Gibco) overnight to remove the epidermis. The dermis was then digested with 2.5 mg/ml collagenase D (Roche) and DNase 1 (Sigma–Aldrich) with rotation at 37°C for 2 h. The cells were harvested after filtration through a 40 μm filter and the lysis of red blood cells. Primary dermal fibroblasts were cultured in DMEM (Gibco) supplemented with 10% FBS (Gibco), GlutaMAX (Gibco) and antibiotic-antimicotic (Gibco) at 37°C in a humidified incubator containing 5% CO_2_. For adipogenesis induction, two-day postconfluent dFBs were cultured in adipocyte differentiation medium (culture medium with 2 μM dexamethasone, 250 μM IBMX, 200 μM indomethacin and 10 μg/ml recombinant human insulin, Sigma–Aldrich). Culture medium with 10 μg/ml recombinant human insulin was used to maintain differentiation.

For *in vitro* studies, live *C*. *albicans* (MOI = 1 or MOI = 10 for yeasts, MOI = 0.1 or MOI = 10 for hyphae) or PBS control was added to postconfluent dFBs that were treated or not with adipocyte differentiation medium. The cells were collected at the indicated time points (4 h or 12 h for RNA and 0.5 h, 1 h, 2 h or 4 h for protein). UV-killed *C*. *albicans* hyphae (MOI = 0.5), β-glucan (250 μg/ml, isolated as previously described [27]) and candidalysin (SIIGIIMGILGNIPQVIQIIMSIVKAFKGNK, synthesized by AtaGenix Laboratories Co., Ltd., Wuhan, China, reconstituted in sterile purified water [52]) were also used in this research. For inhibitor application, dFBs were pretreated with 10 μM U0126 (MEK inhibitor, catalog number S1102, Selleck), 1 μM AZD4547 (FGFR inhibitor, catalog number HY-13330, MedChemExpress), 1 μM PD1530135 (EGFR inhibitor, catalog number S1079, Selleck) or DMSO vehicle control for 2 h before *C*. *albicans* stimulation.

### Lentivirus plasmid construction and transfection

The shRNA targeting *Camp* was designed by GenePharma (Shanghai, China). Mouse dFBs were infected with lentiviral shRNA according to the manufacturer’s instructions. Empty vector was used as a negative control. Twenty-four hours after transfection, culture medium containing lentivirus was removed. The cells were then cultured to 100% confluency for 2 days and transferred to adipocyte differentiation medium to initiate adipocyte differentiation as described above. Conditioned medium was collected during this process, and knockdown efficiencies were determined by western blotting. NC shRNA sequence: 5′-GTTCTCCGAACGTGTCACGT-3′; CAMP shRNA sequence: 5′-TCCGAGCTGTGGATGACTTCA-3′.

### Small interfering RNA transfection

Mouse dFBs in culture were transfected with mouse FGFR1 siRNA (final concentration 12.5 nM) or stable negative control (NC) siRNA (GenePharma). Lipofectamin RNAiMAX (Invitrogen) was used for transfection according to the manufacturer’s instructions. The sense sequence for FGFR1 siRNA is 5′-GCCGUGAAGAUGUUGAAGUTT-3′. The sequence for NC siRNA is 5′-UUCUCCGAACGUGUCACGUTT-3′.

### Histology, immunohistochemistry and immunocytochemistry

Tissue biopsies were embedded in paraffin for hematoxylin-eosin (H&E), PAS and collagen trichrome staining. For Oil red O (ORO) staining, fixed cells were stained with 0.3% Oil red O for 15 mins. For immunofluorescence staining, mouse skin samples were directly embedded in OCT compound. Skin sections were fixed with 4% PFA, permeabilized with 0.2% Triton X-100 and blocked with goat serum before incubation with primary antibodies overnight at 4°C. Secondary antibody staining was performed the next day at room temperature for 1 h, and nuclei were counterstained with DAPI. Human skin sections that were embedded in paraffin were first dewaxed, followed by antigen retrieval prior to blocking and incubation with antibodies. For BODIPY staining, 1 μM BODIPY buffer solution was added to fixed tissue sections or live cells for 30 min. All images were captured with confocal laser scanning microscopy (Olympus FV1000). Specific antibodies are shown in S3 Table.

### RNA extraction and quantitative reverse transcription PCR (qRT-PCR)

Total cellular RNA was isolated with TRIzol reagent (Invitrogen, Carlsbad, CA, USA) according to the manufacturer’s procedures. A total of 1000 ng of RNA was reverse transcribed to cDNA using HiScript III RT SuperMix for qPCR (+gDNA wiper) (Vazyme). Quantitative real-time PCR was performed using SYBR Green Pro Taq HS Premix (Accurate Biology, Changsha, China) on a LightCycler 480 System (Roche). The *Tbp* gene (TATA-Box Binding Protein) was used as a housekeeping gene. All the primers are listed in S2 Table.

### Protein extraction and western blotting

Cultured cells or mouse skin biopsies were lysed with RIPA buffer (Beyotime, Shanghai, China) containing phosphatase and protease inhibitors (Roche, Mannheim, Germany). After sonication and centrifugation at 12000 rpm for 15 min, the supernatants were collected. A Pierce BCA Protein Assay Kit (Thermo Fisher Scientific, 23227) was used to quantify the protein concentrations. Protein (40 μg/μl) was boiled with SDS–PAGE loading buffer (Beyotime, Shanghai, China) and added to PAGE Precast Protein Gels (Smart-Lifesciences, Changzhou, China). In some experiments, 20 μl of conditioned medium was used for western blotting analyses. The proteins were then transferred to a PVDF membrane (Bio-Rad), blocked with 5% nonfat milk and incubated overnight at 4°C with primary antibodies. After incubation with secondary antibodies, the membranes were visualized with an ECL kit (Bio-Rad). A rabbit anti-CRAMP antibody was generously provided by Professor Gallo from University of California San Diego as previously described [15, 26]. Other antibodies used in this study are shown in S3 Table.

### ELISA

Mouse Camp ELISA Kit was purchased from CUSABIO (catalog number CSB-E15061m). Culture supernatants were collected and CAMP concentrations were determined according to the manufacturer’s procedures.

### *In vitro* antimicrobial assay

Antibiotic-free conditioned medium was collected from differentiating dermal fibroblasts as previously described [15], mixed with 10^4^ CFU *C*. *albicans* in 96-well plates, and incubated at 37°C for 24 h. The optical density at 600 nm (OD600) was measured every 6 hours. CAMP neutralizing antibody (Hycult Biotech, catalog number HM2070) was added at 5 μg/ml for cathelicidin neutralization. Mouse IgG (NI03, Sigma–Aldrich) was used as a control. CFUs were also counted by plating serial dilutions on SDA.

### LDH release assay

The activity of LDH in culture supernatants was measured using a LDH cytotoxicity assay kit (Beyotime, Shanghai, China). The manufacturer’s instructions were followed and samples were measured for LDH immediately after collection.

### Flow cytometry and analysis (FACS)

Dermal fibroblasts were stimulated with *C*. *albicans*, washed with PBS and then incubated with antibodies on ice for 30 min. The following antibodies were used: PE anti-THY1 (Biolegend, 105308), APC anti-PDGFRA (eBioscience, 17-1401-81), PE anti-TLR2 (Biolegend, 153004), PE/Cyanine7 anti-TLR4 (Biolegend, 117610) and APC anti-Dectin-1 (Biolegend, 144306). To assess Dectin-1 expression, mouse bone marrow-derived macrophages were used as a positive control. FACS analysis was performed on the BD FACSVers and analyzed by FlowJo V10 software.

### Bone marrow-derived macrophage (BMDM) isolation and culture

Bone marrow cells were isolated as described before [53]. In brief, femurs and tibias from C57BL/6 mice were separated and flushed to obtain bone marrow cells. After filtration and lysis of red blood cells, cells were then cultured in differentiation medium (DMEM containing 10% FBS, 50 ng/ml mouse M-CSF (Meltenyi, Bergisch Gladbach, Germany) and antibiotic-antimycotic) for 7 days. BMDMs were stimulated with the same amount of *C*. *albicans* as it on dermal fibroblasts.

### Statistical analyses

Experiments were repeated at least 3 times, and similar results were obtained. GraphPad Prism 9.0 software was used for statistical analyses. Student’s unpaired two-tailed t test (for two groups) or one-way ANOVA multiple comparison test (for three or more groups) was used to calculate statistical significance as indicated (*P < 0.05, **P < 0.01 and ***P < 0.001).

## Supporting information

S1 Table Information of patients with cutaneous *Candida* granulomas. (DOCX)

S2 Table Primers for qRT-PCR. (DOCX)

S3 Table List of antibodies and dyes used in the study. (DOCX)

S1 Fig (related to Fig 2): *C*. *albicans* infection increases dermal adipocyte numbers. (A) Hematoxylin and eosin of mouse skin infected with *C*. *albicans* hyphae (3 day after injection). (B) Periodic acid–Schiff (PAS) staining of *C*. *albicans* in mouse skin infected with *C*. *albicans* yeasts (1 day after injection). (C) Representative images of mouse skin lesions after intradermally injected with PBS control or *C*. *albicans* yeasts or hyphae on the 1^st^ day or 3^rd^ day after injection. Scale bars, 1 mm. (D and E) Mouse skin were intradermally injected with PBS control or *C*. *albicans* yeasts or hyphae, skin samples were collected 1 day after infection. (D) Hematoxylin and eosin (top) or BODIPY staining (bottom) of mouse skin. Nuclei were stained with DAPI. Scale bars, 100 μm. (E) DWAT thickness of mouse skin (n = 6/group). (F) Collagen trichrome staining of mouse skin collected 3 days after infection. Scale bar, 100 μm. All error bars indicate mean ± SD. *P < 0.05, **P < 0.01, ***P < 0.001 (one-way ANOVA). dWAT, dermal white adipose tissue; PBS, phosphate buffered saline; PHA, phalloidin.
(TIF)

S2 Fig (related to Fig 3): Adipogenesis-related PPARγ activity in infected skin may be crucial to counteract *C*. *albicans* infection. Mice were intraperitoneally injected with BADGE or GW9662 or DMSO vehicle control daily starting 1 day before *C*. *albicans* infection, skin samples were collected 3 days after infection. (A) *Pref1* mRNA expression in skin tissue as indicated (n = 5/group). (B) *16S rRNA* mRNA expression in skin tissue as indicated (n = 6/group). (C) *16S rRNA* mRNA expression in *C*. *albicans* yeasts or *S*. *aureus* (*S*.*A*.)-infected mouse skin. PBS was used as control (n = 3/group). For *S*. *aureus* infection, a total of 75 μl of 1×10^7^/ml log-phase *S*. *aureus* (ATCC25923, preserved in the China Medical Fungus Culture Collection Center) was prepared in PBS and intradermally injected. (D) GW9662 increased susceptibility to *C*. *albicans* skin infection as shown by increased CFU count in skin lesion (n = 5/group). (E) Skin images or skin lesion size quantification of *C*. *albicans*-infected mouse skin after treatment of BADGE or GW9662 or DMSO control (n = 8/group). Scale bars, 1 mm. All error bars indicate mean ± SD. *P < 0.05, **P < 0.01, ***P < 0.001 (Unpaired t test was used in A, B, D and E, one-way ANOVA multiple comparison test was used to determine statistical significance in C). *S*. *aureus*, *Staphylococcus aureus*; CFU, colony-forming unit; NS, not significant.
(TIF)

S3 Fig (related to Fig 4): Increase in cathelicidin expression after *C*. *albicans* stimulation or preadipocyte adipogenesis. (A) Western blotting analysis of cathelicidin (CAMP) in mouse skin 3 days after intradermally injection with PBS control or *C*. *albicans*. (B) Neonatal dermal fibroblasts were cultured to reach 100% confluence for 2 days (preadipocytes, pAds), then stimulated with PBS control (Ctrl), UV-killed hyphae or β-glucan for 4 h. Western blotting analysis of CAMP protein level. (C) Phase-contrast images of undifferentiated pAds (Undif) or differentiating adipocytes (AD) during adipocyte differentiation. Lipid production was shown by BODIPY or Oil-Red-O staining. Scale bar, 100 μm. (D) OD600 of *C*. *albicans* cultured in CM from culture medium (Ctrl), Undif or AD. All error bars indicate mean ± SD. CM, conditioned medium.
(TIF)

S4 Fig (related to Fig 5): *C*. *albicans*-induced *Camp* expression in pAds is not via TLRs or dectin-1. (A) Loss of *Myd88* expression in primary dermal fibroblasts isolated from *Myd88*^*−/−*^ mice (n = 4/group). (B) *Camp* expression was not suppressed in *Myd88*^*−/−*^ pAds stimulated with *C*. *albicans* yeasts or hyphae at 12 h. (C and D) Western blotting analysis of TLR2 and TLR4 in pAds stimulated with live hyphae (C) or UV-killed hyphae or β-glucan (D). Equal volume of PBS was used as control (Ctrl). (E–G) Mouse pAds (identified by THY1 or PDGFRA) or bone marrow-derived macrophages (BMDMs) were incubated with live or UV-killed *C*. *albicans* or PBS control for 6 hours. The expression of dectin-1 was detected by flow cytometry. Flow cytometry histograms (E) or the percentages (F) or MFI (G) of dectin-1 were calculated (n = 3/group). All error bars indicate mean ± SD. *P < 0.05, **P < 0.01, ***P < 0.001 (Unpaired t test was used in A, one-way ANOVA multiple comparison test was used to determine statistical significance in B, F and G). NS, not significant; TLR, Toll-like receptor; WT, wild type.
(TIF)

S5 Fig (related to Fig 5): Protein expression in pAds after *C*. *albicans* stimulation. (A) Western blotting analysis of phosphorylated FGFR1 and FRS2-α in pAds incubated with PBS control (Ctrl) or UV-killed hyphae. (B) Immunostaining showed elevated phospho-FRS2-α (red) in mouse skin lesions three days after intradermally injection with *C*. *albicans* yeasts. Scale bar, 100 μm. (C and D) Western blotting analysis of EGFR phosphorylation and quantification in pAds incubated with PBS control or *C*. *albicans* live hyphae (C) or UV-killed hyphae (D). (E and F) Western blotting analysis of protein levels in pAds stimulated with live hyphae (E) or UV-killed hypha or β-glucan (F). Equal volume of PBS was used as control. All error bars indicate mean ± SD. *P < 0.05, **P < 0.01, ***P < 0.001 (one-way ANOVA). EGFR, epidermal growth factor receptor; FGFR, fibroblast growth factor receptor; FRS2, FGFR substrate 2.
(TIF)

S6 Fig (related to Fig 6): Inhibiting MEK-ERK signaling suppresses *C*. *albicans*-induced cathelicidin production by pAds and candidalysin may not be the key driver of FGFR-mediated cathelicidin production in pAds. (A) U0126 application attenuated *Camp* mRNA expression in pAds stimulated with β-glucan for 12 h. (B) Mouse pAds were pretreated with U0126, AZD4547 or vehicle control (DMSO) for 2 h and then stimulated with PBS control (Ctrl) or β-glucan for 4 h. Western blotting analysis of phosphorylated FRS2-α, AKT or ERK1/2. (C) Mice were intradermally injected daily with U0126 or vehicle control (DMSO) starting 20 min before infection with *C*. *albicans*. Skin samples were collected 3 days after infection. Immunostaining showed suppressed colocalization of CAMP (red) and PREF1 (green) in infected mouse skin after treatment with U0126. (D) *Camp* mRNA expression in pAds after stimulation with ddH_2_O (Ctrl) or candidalysin for 1 day. (E) Western blotting analysis of protein levels in pAds stimulated with candidalysin for 4 h. (F) Western blotting analysis of EGFR phosphorylation in pAds incubated with candidalysin. (G) *Camp* mRNA expression in pAds after pretreatment with U0126, AZD4547 or vehicle control (DMSO) for 2 h and then stimulated with candidalysin (30 μM) for 12 h. (H) Western blotting analysis of phosphorylated ERK1/2 in pAds after pretreatment with AZD4547 and then stimulated with candidalysin (30 μM) for 4 h. All error bars indicate mean ± SD. *P < 0.05, **P < 0.01, ***P < 0.001 (one-way ANOVA). ddH2O, deionized distilled water.
(TIF)
