# Supplementary material for: Antimicrobial peptide-producing dermal preadipocytes defend against Candida albicans skin infection via the FGFR-MEK-ERK pathway
Source: PLoS Pathog. 2023 Nov 30;19(11):e1011754. doi: 10.1371/journal.ppat.1011754 (PMC10688742; doi:10.1371/journal.ppat.1011754)
Supplement: S1 Table — (DOCX) [file ppat.1011754.s001.docx]

**S1 Table**

Information of patients with cutaneous *Candida* granulomas.

| Number | Gender | Age | Site of lesions | Underlying health conditions |
| --- | --- | --- | --- | --- |
| 1  2 | Male  Female | 51  50 | Extremity  Face | Unidentified  None |
| 3  4 | Female  Female | 63  37 | Trunk  Extremity | None  Focal segmental glomerulosclerosis, treated with prednisone and tacrolimus |
