# Supplementary material for: Antimicrobial peptide-producing dermal preadipocytes defend against Candida albicans skin infection via the FGFR-MEK-ERK pathway
Source: PLoS Pathog. 2023 Nov 30;19(11):e1011754. doi: 10.1371/journal.ppat.1011754 (PMC10688742; doi:10.1371/journal.ppat.1011754)
Supplement: S2 Table — (DOCX) [file ppat.1011754.s002.docx]

**S2 Table**

Primers for qRT-PCR.

| Gene |  | Sequence 5ʹ to 3ʹ (mouse) |
| --- | --- | --- |
| *Tbp*  *Camp*  *Pref1*  *Cebpb*  *Pparg1*  *Col1a1* | Forward  Reverse  Foward  Reverse  Foward  Reverse  Foward  Reverse  Foward  Reverse  Foward  Reverse | CCTTGTACCCTTCACCAATGAC  ACAGCCAAGATTCACGGTAGA  CAAGGAACAGGGGGTGG  TCCGGCTGAGGTACAAGTTT  TGGCTGGGACGGGAAATTC  CACGCAAGTTCCATTGTTGGC  CAAGAGCCGCGACAAGGCCA  CTCGCGACAGCTGCTCCACC  AAGAAGCGGTGAACCACTGA  GGAATGCGAGTGGTCTTCCA  GCTCCTCTTAGGGGCCACT  ATTGGGGACCCTTAGGCCAT |
| *Myd88*  *16S rRNA* | Foward  Reverse  Foward  Reverse | CTATCGCTGTTCTTGAACCCTC  TCCACTCTGGCCACCTGTAA  ACTCCTACGGGAGGCAGCAGT  GTATTACCGCGGCTGCTGGCAC |
