# Supplementary material for: Antimicrobial peptide-producing dermal preadipocytes defend against Candida albicans skin infection via the FGFR-MEK-ERK pathway
Source: PLoS Pathog. 2023 Nov 30;19(11):e1011754. doi: 10.1371/journal.ppat.1011754 (PMC10688742; doi:10.1371/journal.ppat.1011754)
Supplement: S3 Table — (DOCX) [file ppat.1011754.s003.docx]

**S3 Table**

List of antibodies and dyes used in the study.

| **Reagents** | **Source** | **Cat No.** |
| --- | --- | --- |
| Rabbit anti-Cathelicidin | Abcam | ab180760 |
| Mouse anti-DLK-1 | Abcam | ab119930 |
| Rabbit anti-phospho-C/EBPβ (Thr235) | Cell Signaling | #3084 |
| Rabbit anti-PPARγ | Cell Signaling | #2435 |
| Rabbit anti-Perilipin-1 | Cell Signaling | #9349 |
| Mouse anti-A-FABP | Santa Cruz | sc-271529 |
| Rabbit anti-phospho-Erk1/2 | Cell Signaling | #4377 |
| Rabbit anti-Erk1/2 | Cell Signaling | #4695 |
| Rabbit anti-phospho-FGFR1 (Y654) | Abcam | ab59194 |
| Rabbit anti-FGF Receptor 1 | Cell Signaling | #9740 |
| Rabbit anti-phospho-FRS2-α (Tyr196) | Cell Signaling | #3864 |
| Rabbit anti-phospho-EGF Receptor (Tyr1068) | Cell Signaling | #3777 |
| Rabbit anti-EGF Receptor | Cell Signaling | #4267 |
| Rabbit anti-TLR2 | Cell Signaling | #13744 |
| Rabbit anti-TLR4 | Abcam | ab13556 |
| Rabbit anti-phospho-Stat3 (Tyr705) | Cell Signaling | #9145 |
| Rabbit anti-Stat3 | Cell Signaling | #4904 |
| Rabbit anti-phospho-Akt (Ser473) | Cell Signaling | #4060 |
| Rabbit anti-Akt (pan) | Cell Signaling | #4691 |
| Rabbit anti-phospho-p38 MAPK (Thr180/Tyr182) | Cell Signaling | #4511 |
| Rabbit anti-p38 MAPK | Cell Signaling | #9212 |
| Rabbit anti-phospho-SAPK/JNK (Thr183/Tyr185) | Cell Signaling | #4668 |
| Rabbit anti-SAPK/JNK | Cell Signaling | #9252 |
| Rabbit anti-phospho-IκBα (Ser32) | Cell Signaling | #2859 |
| Rabbit anti-IκBα | Cell Signaling | #4812 |
| Rabbit anti-GAPDH | Cell Signaling | #2118 |
| Rabbit anti-β-Tubulin | Cell Signaling | #2146 |
| Bodipy | ThermoFisher Scientific | D3922 |
| Phalloidin-iFluor 647 Reagent | Abcam | ab176759 |
| DAPI | Beyotime | C1006 |
