# Supplementary figures and images for: Antimicrobial peptide-producing dermal preadipocytes defend against Candida albicans skin infection via the FGFR-MEK-ERK pathway
Source: PLoS Pathog. 2023 Nov 30;19(11):e1011754. doi: 10.1371/journal.ppat.1011754 (PMC10688742; doi:10.1371/journal.ppat.1011754)

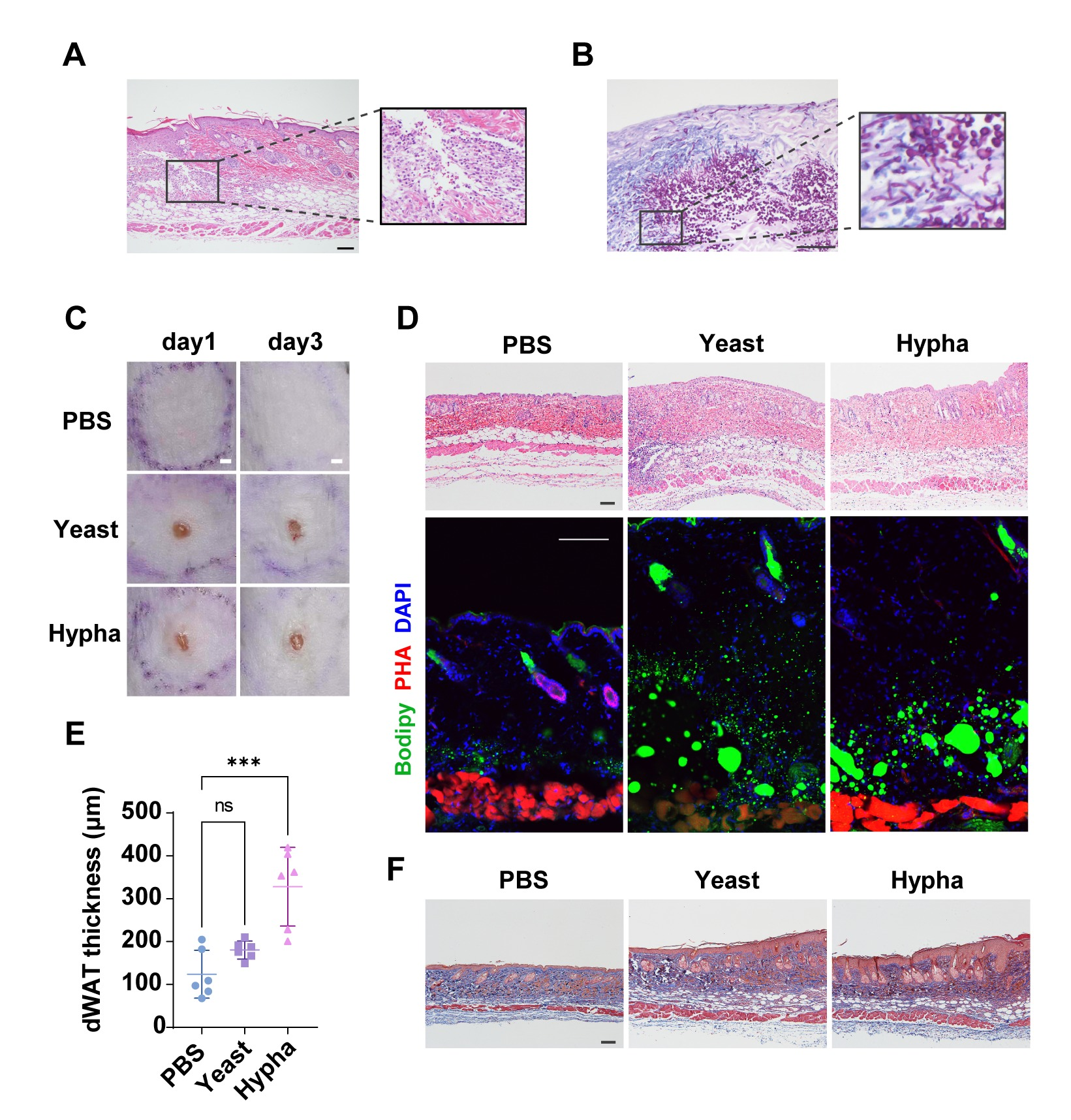

Supplement: S1 Fig — (A) Hematoxylin and eosin of mouse skin infected with C. albicans hyphae (3 day after injection). (B) Periodic acid–Schiff (PAS) staining of C. albicans in mouse skin infected with C. albicans yeasts (1 day after injection). (C) Representative images of mouse skin lesions after intradermally injected with PBS control or C. albicans yeasts or hyphae on the 1st day or 3rd day after injection. Scale bars, 1 mm. (D and E) Mouse skin were intradermally injected with PBS control or C. albicans yeasts or hyphae, skin samples were collected 1 day after infection. (D) Hematoxylin and eosin (top) or BODIPY staining (bottom) of mouse skin. Nuclei were stained with DAPI. Scale bars, 100 μm. (E) DWAT thickness of mouse skin (n = 6/group). (F) Collagen trichrome staining of mouse skin collected 3 days after infection. Scale bar, 100 μm. All error bars indicate mean ± SD. *P < 0.05, **P < 0.01, ***P < 0.001 (one-way ANOVA). dWAT, dermal white adipose tissue; PBS, phosphate buffered saline; PHA, phalloidin. (TIF) [file ppat.1011754.s004.tif]

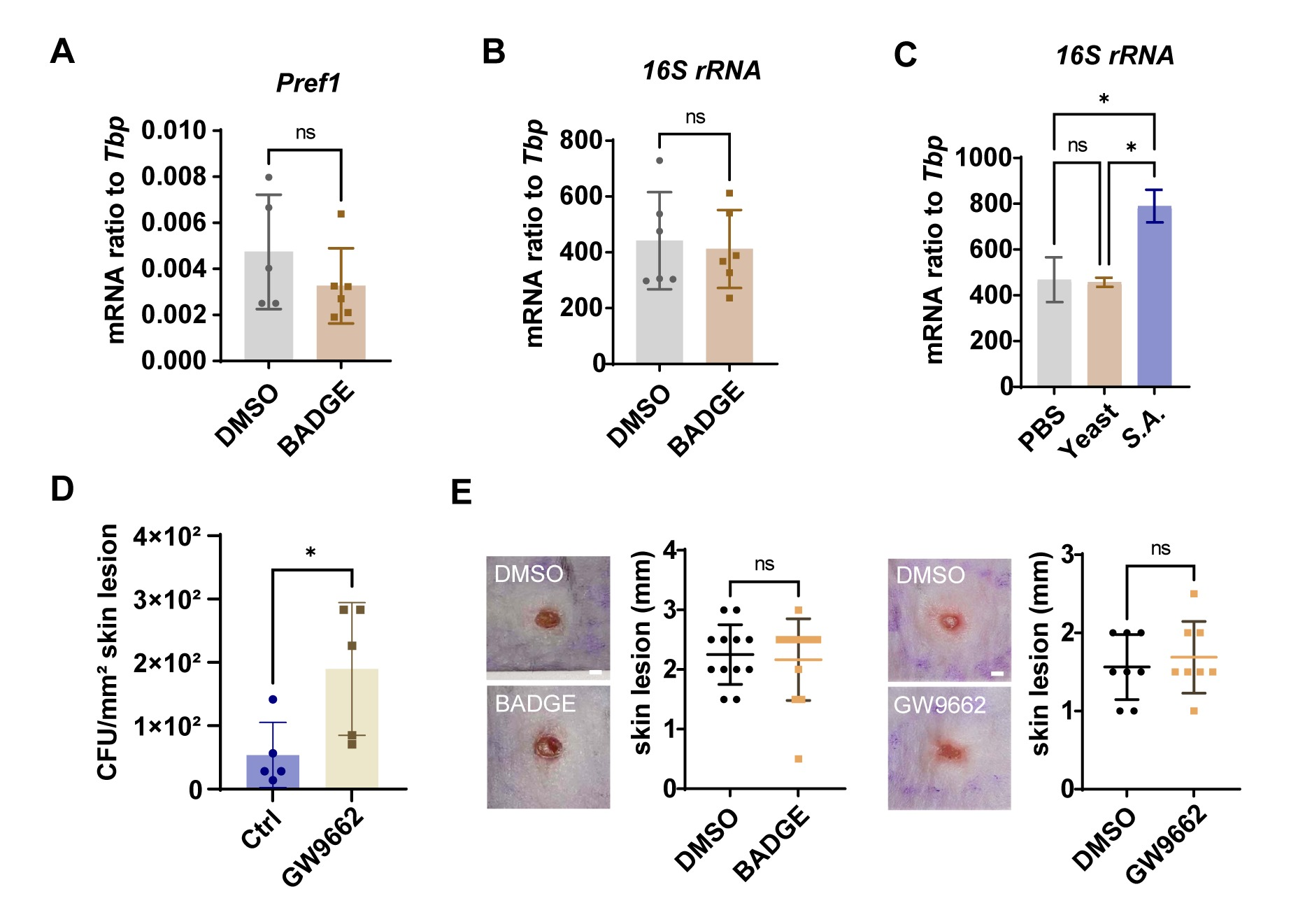

Supplement: S2 Fig — Mice were intraperitoneally injected with BADGE or GW9662 or DMSO vehicle control daily starting 1 day before C. albicans infection, skin samples were collected 3 days after infection. (A) Pref1 mRNA expression in skin tissue as indicated (n = 5/group). (B) 16S rRNA mRNA expression in skin tissue as indicated (n = 6/group). (C) 16S rRNA mRNA expression in C. albicans yeasts or S. aureus (S.A.)-infected mouse skin. PBS was used as control (n = 3/group). For S. aureus infection, a total of 75 μl of 1×10^7^/ml log-phase S. aureus (ATCC25923, preserved in the China Medical Fungus Culture Collection Center) was prepared in PBS and intradermally injected. (D) GW9662 increased susceptibility to C. albicans skin infection as shown by increased CFU count in skin lesion (n = 5/group). (E) Skin images or skin lesion size quantification of C. albicans-infected mouse skin after treatment of BADGE or GW9662 or DMSO control (n = 8/group). Scale bars, 1 mm. All error bars indicate mean ± SD. *P < 0.05, **P < 0.01, ***P < 0.001 (Unpaired t test was used in A, B, D and E, one-way ANOVA multiple comparison test was used to determine statistical significance in C). S. aureus, Staphylococcus aureus; CFU, colony-forming unit; NS, not significant. (TIF) [file ppat.1011754.s005.tif]

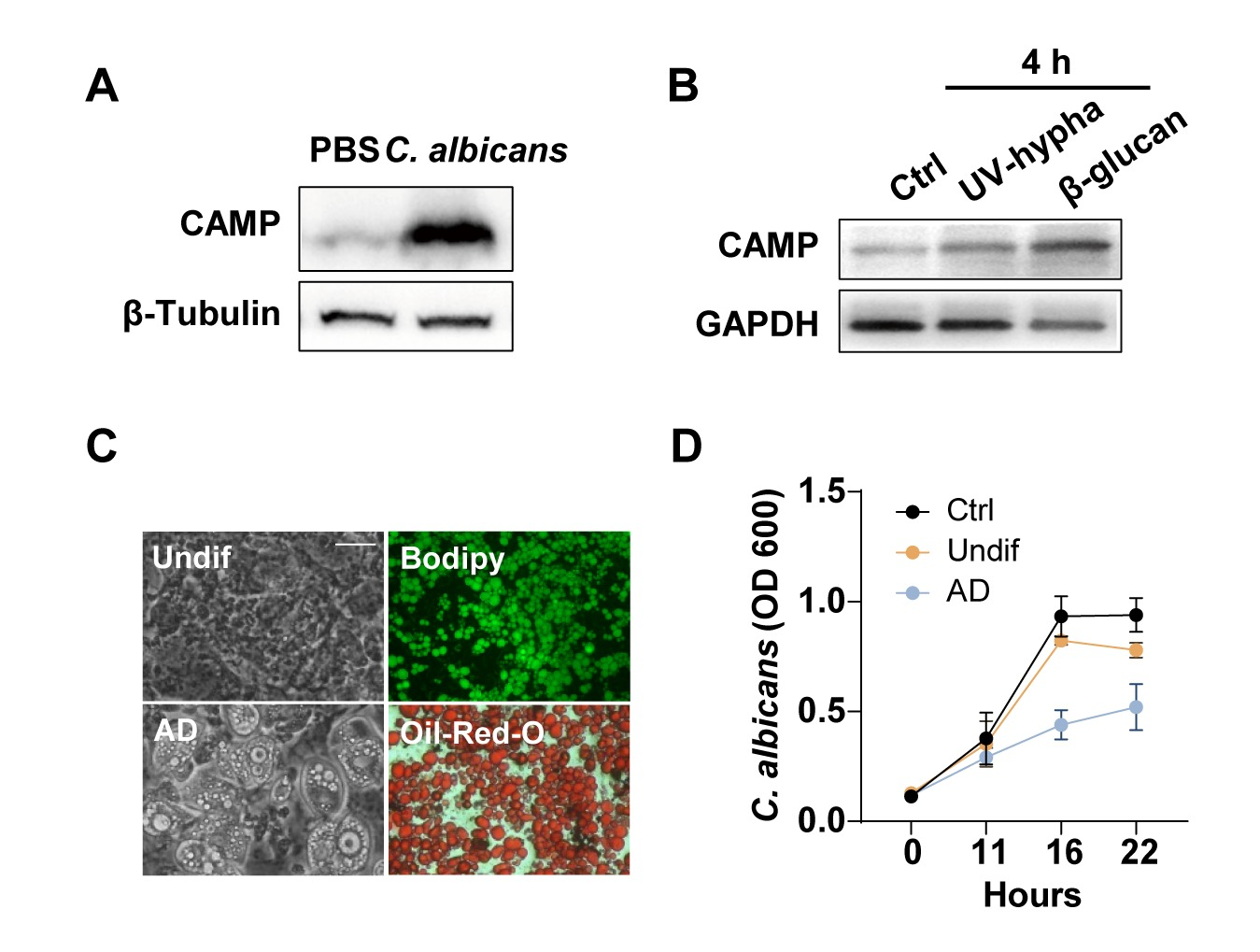

Supplement: S3 Fig — (A) Western blotting analysis of cathelicidin (CAMP) in mouse skin 3 days after intradermally injection with PBS control or C. albicans. (B) Neonatal dermal fibroblasts were cultured to reach 100% confluence for 2 days (preadipocytes, pAds), then stimulated with PBS control (Ctrl), UV-killed hyphae or β-glucan for 4 h. Western blotting analysis of CAMP protein level. (C) Phase-contrast images of undifferentiated pAds (Undif) or differentiating adipocytes (AD) during adipocyte differentiation. Lipid production was shown by BODIPY or Oil-Red-O staining. Scale bar, 100 μm. (D) OD600 of C. albicans cultured in CM from culture medium (Ctrl), Undif or AD. All error bars indicate mean ± SD. CM, conditioned medium. (TIF) [file ppat.1011754.s006.tif]

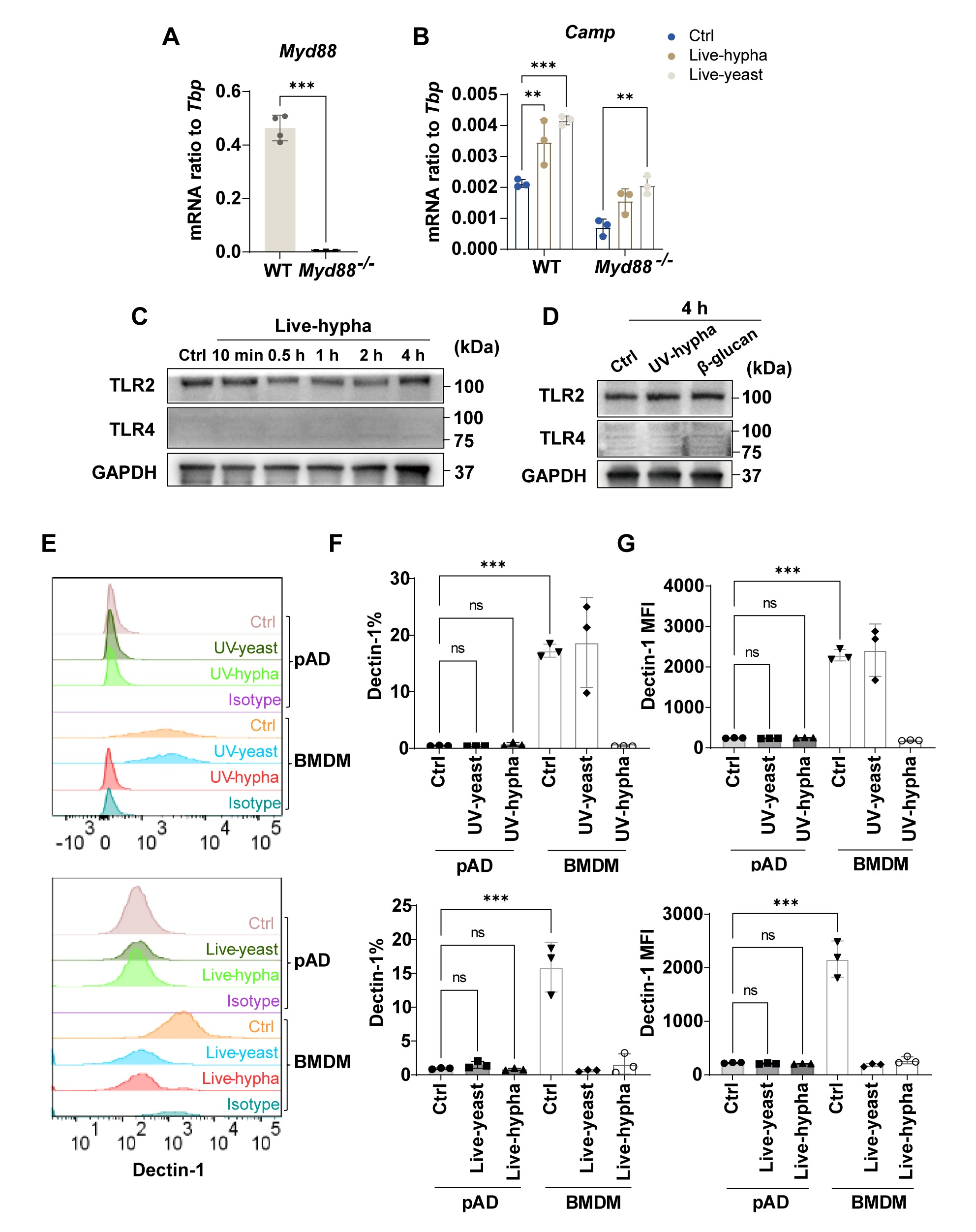

Supplement: S4 Fig — (A) Loss of Myd88 expression in primary dermal fibroblasts isolated from Myd88−/− mice (n = 4/group). (B) Camp expression was not suppressed in Myd88−/− pAds stimulated with C. albicans yeasts or hyphae at 12 h. (C and D) Western blotting analysis of TLR2 and TLR4 in pAds stimulated with live hyphae (C) or UV-killed hyphae or β-glucan (D). Equal volume of PBS was used as control (Ctrl). (E–G) Mouse pAds (identified by THY1 or PDGFRA) or bone marrow-derived macrophages (BMDMs) were incubated with live or UV-killed C. albicans or PBS control for 6 hours. The expression of dectin-1 was detected by flow cytometry. Flow cytometry histograms (E) or the percentages (F) or MFI (G) of dectin-1 were calculated (n = 3/group). All error bars indicate mean ± SD. *P < 0.05, **P < 0.01, ***P < 0.001 (Unpaired t test was used in A, one-way ANOVA multiple comparison test was used to determine statistical significance in B, F and G). NS, not significant; TLR, Toll-like receptor; WT, wild type. (TIF) [file ppat.1011754.s007.tif]

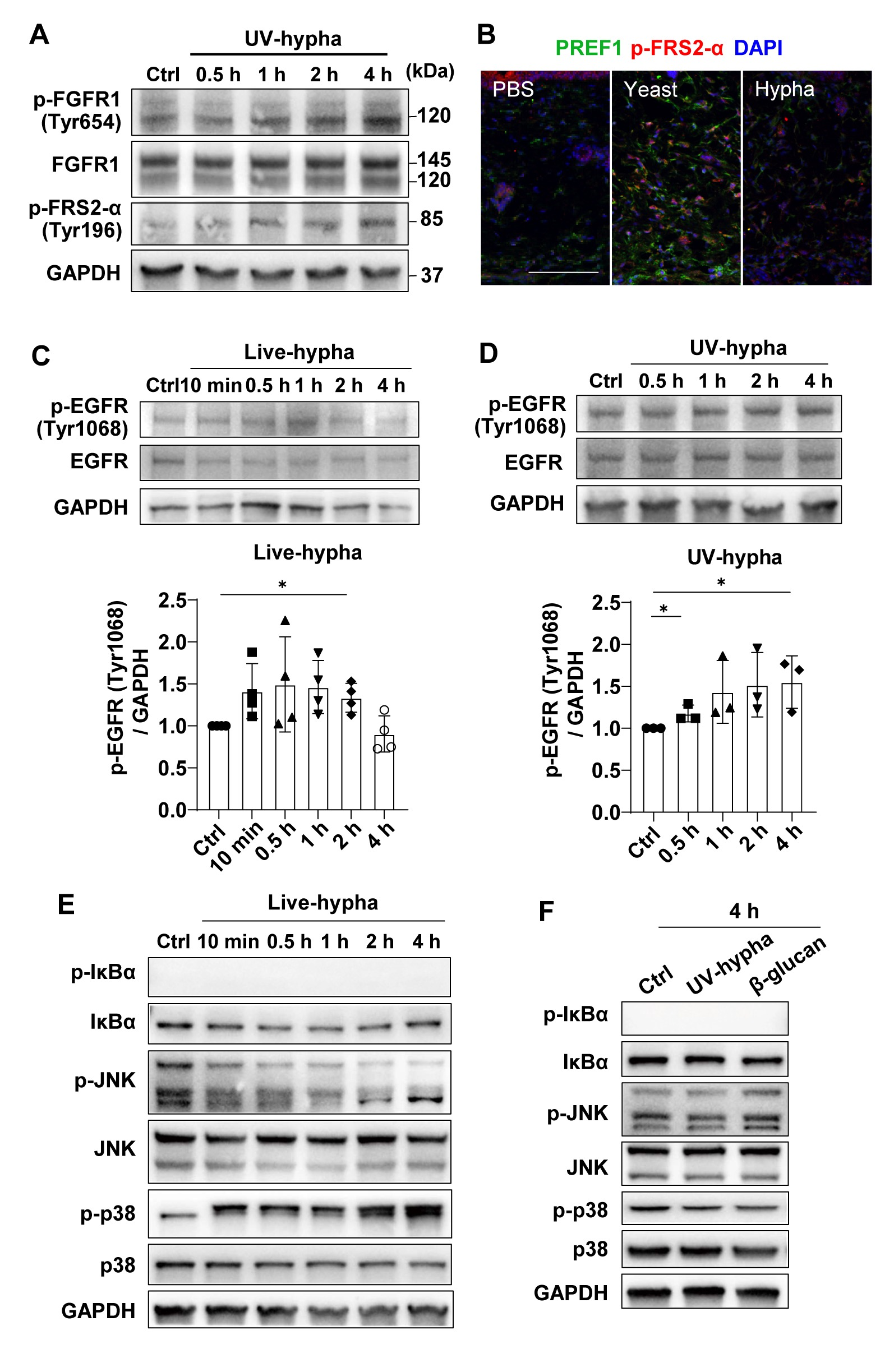

Supplement: S5 Fig — (A) Western blotting analysis of phosphorylated FGFR1 and FRS2-α in pAds incubated with PBS control (Ctrl) or UV-killed hyphae. (B) Immunostaining showed elevated phospho-FRS2-α (red) in mouse skin lesions three days after intradermally injection with C. albicans yeasts. Scale bar, 100 μm. (C and D) Western blotting analysis of EGFR phosphorylation and quantification in pAds incubated with PBS control or C. albicans live hyphae (C) or UV-killed hyphae (D). (E and F) Western blotting analysis of protein levels in pAds stimulated with live hyphae (E) or UV-killed hypha or β-glucan (F). Equal volume of PBS was used as control. All error bars indicate mean ± SD. *P < 0.05, **P < 0.01, ***P < 0.001 (one-way ANOVA). EGFR, epidermal growth factor receptor; FGFR, fibroblast growth factor receptor; FRS2, FGFR substrate 2. (TIF) [file ppat.1011754.s008.tif]

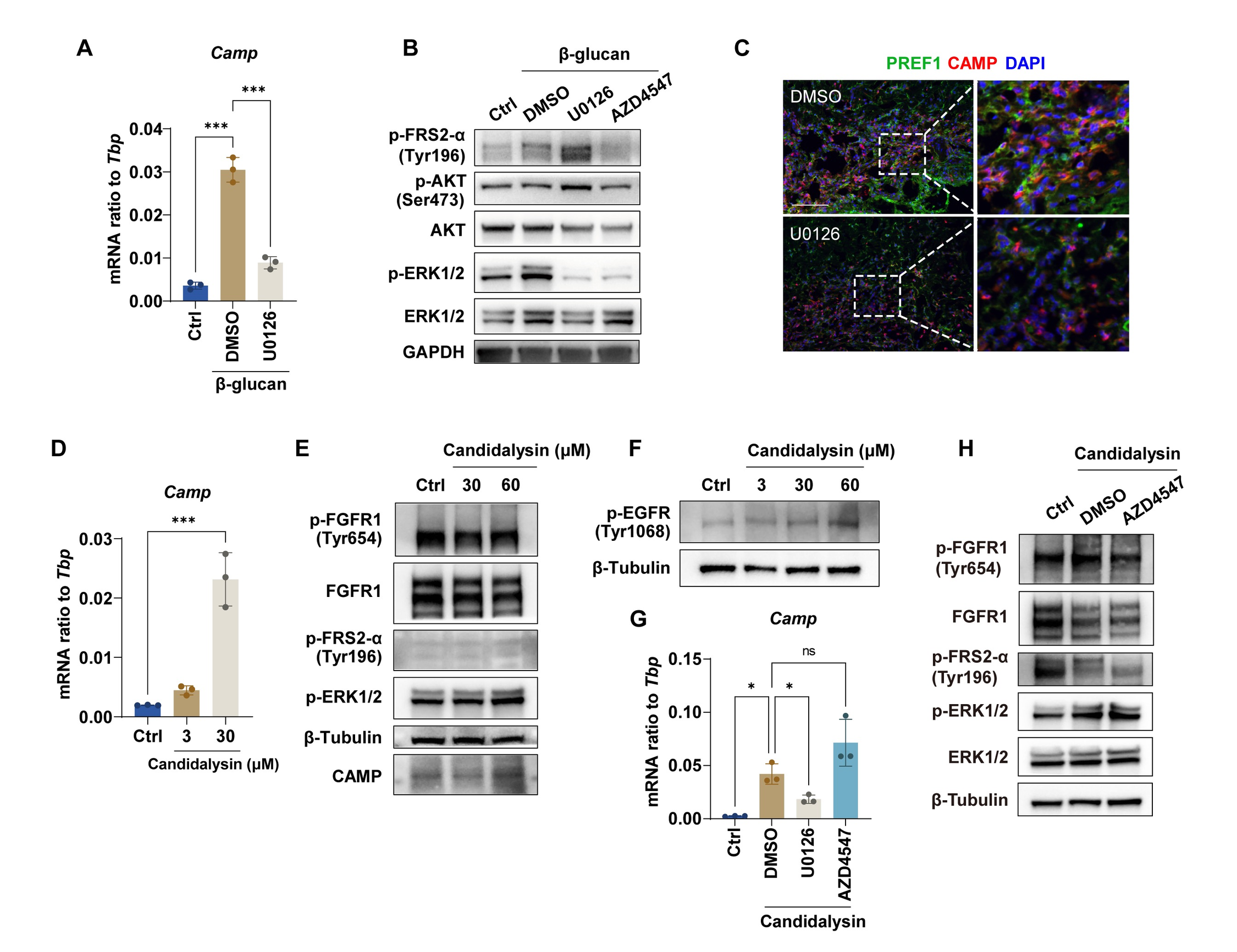

Supplement: S6 Fig — (A) U0126 application attenuated Camp mRNA expression in pAds stimulated with β-glucan for 12 h. (B) Mouse pAds were pretreated with U0126, AZD4547 or vehicle control (DMSO) for 2 h and then stimulated with PBS control (Ctrl) or β-glucan for 4 h. Western blotting analysis of phosphorylated FRS2-α, AKT or ERK1/2. (C) Mice were intradermally injected daily with U0126 or vehicle control (DMSO) starting 20 min before infection with C. albicans. Skin samples were collected 3 days after infection. Immunostaining showed suppressed colocalization of CAMP (red) and PREF1 (green) in infected mouse skin after treatment with U0126. (D) Camp mRNA expression in pAds after stimulation with ddH2O (Ctrl) or candidalysin for 1 day. (E) Western blotting analysis of protein levels in pAds stimulated with candidalysin for 4 h. (F) Western blotting analysis of EGFR phosphorylation in pAds incubated with candidalysin. (G) Camp mRNA expression in pAds after pretreatment with U0126, AZD4547 or vehicle control (DMSO) for 2 h and then stimulated with candidalysin (30 μM) for 12 h. (H) Western blotting analysis of phosphorylated ERK1/2 in pAds after pretreatment with AZD4547 and then stimulated with candidalysin (30 μM) for 4 h. All error bars indicate mean ± SD. *P < 0.05, **P < 0.01, ***P < 0.001 (one-way ANOVA). ddH2O, deionized distilled water. (TIF) [file ppat.1011754.s009.tif]
